# Search for minimal supersymmetric standard model Higgs Bosons *H* / *A* and for a $Z^{\prime }$ boson in the $\tau \tau$ final state produced in *pp* collisions at $\sqrt{s}= 13$ TeV with the ATLAS detector

**DOI:** 10.1140/epjc/s10052-016-4400-6

**Published:** 2016-10-27

**Authors:** M. Aaboud, G. Aad, B. Abbott, J. Abdallah, O. Abdinov, B. Abeloos, R. Aben, O. S. AbouZeid, N. L. Abraham, H. Abramowicz, H. Abreu, R. Abreu, Y. Abulaiti, B. S. Acharya, S. Adachi, L. Adamczyk, D. L. Adams, J. Adelman, S. Adomeit, T. Adye, A. A. Affolder, T. Agatonovic-Jovin, J. Agricola, J. A. Aguilar-Saavedra, S. P. Ahlen, F. Ahmadov, G. Aielli, H. Akerstedt, T. P. A. Åkesson, A. V. Akimov, G. L. Alberghi, J. Albert, S. Albrand, M. J. Alconada Verzini, M. Aleksa, I. N. Aleksandrov, C. Alexa, G. Alexander, T. Alexopoulos, M. Alhroob, B. Ali, M. Aliev, G. Alimonti, J. Alison, S. P. Alkire, B. M. M. Allbrooke, B. W. Allen, P. P. Allport, A. Aloisio, A. Alonso, F. Alonso, C. Alpigiani, A. A. Alshehri, M. Alstaty, B. Alvarez Gonzalez, D. Álvarez Piqueras, M. G. Alviggi, B. T. Amadio, K. Amako, Y. Amaral Coutinho, C. Amelung, D. Amidei, S. P. Amor Dos Santos, A. Amorim, S. Amoroso, G. Amundsen, C. Anastopoulos, L. S. Ancu, N. Andari, T. Andeen, C. F. Anders, G. Anders, J. K. Anders, K. J. Anderson, A. Andreazza, V. Andrei, S. Angelidakis, I. Angelozzi, P. Anger, A. Angerami, F. Anghinolfi, A. V. Anisenkov, N. Anjos, A. Annovi, C. Antel, M. Antonelli, A. Antonov, F. Anulli, M. Aoki, L. Aperio Bella, G. Arabidze, Y. Arai, J. P. Araque, A. T. H. Arce, F. A. Arduh, J-F. Arguin, S. Argyropoulos, M. Arik, A. J. Armbruster, L. J. Armitage, O. Arnaez, H. Arnold, M. Arratia, O. Arslan, A. Artamonov, G. Artoni, S. Artz, S. Asai, N. Asbah, A. Ashkenazi, B. Åsman, L. Asquith, K. Assamagan, R. Astalos, M. Atkinson, N. B. Atlay, K. Augsten, G. Avolio, B. Axen, M. K. Ayoub, G. Azuelos, M. A. Baak, A. E. Baas, M. J. Baca, H. Bachacou, K. Bachas, M. Backes, M. Backhaus, P. Bagiacchi, P. Bagnaia, Y. Bai, J. T. Baines, O. K. Baker, E. M. Baldin, P. Balek, T. Balestri, F. Balli, W. K. Balunas, E. Banas, Sw. Banerjee, A. A. E. Bannoura, L. Barak, E. L. Barberio, D. Barberis, M. Barbero, T. Barillari, M-S Barisits, T. Barklow, N. Barlow, S. L. Barnes, B. M. Barnett, R. M. Barnett, Z. Barnovska-Blenessy, A. Baroncelli, G. Barone, A. J. Barr, L. Barranco Navarro, F. Barreiro, J. Baarreiro Guimrães da Costa, R. Bartoldus, A. E. Barton, P. Bartos, A. Basalaev, A. Bassalat, R. L. Bates, S. J. Batista, J. R. Batley, M. Battaglia, M. Bauce, F. Bauer, H. S. Bawa, J. B. Beacham, M. D. Beattie, T. Beau, P. H. Beauchemin, P. Bechtle, H. P. Beck, K. Becker, M. Becker, M. Beckingham, C. Becot, A. J. Beddall, A. Beddall, V. A. Bednyakov, M. Bedognetti, C. P. Bee, L. J. Beemster, T. A. Beermann, M. Begel, J. K. Behr, C. Belanger-Champagne, A. S. Bell, G. Bella, L. Bellagamba, A. Bellerive, M. Bellomo, K. Belotskiy, O. Beltramello, N. L. Belyaev, O. Benary, D. Benchekroun, M. Bender, K. Bendtz, N. Benekos, Y. Benhammou, E. Benhar Noccioli, J. Benitez, D. P. Benjamin, J. R. Bensinger, S. Bentvelsen, L. Beresford, M. Beretta, D. Berge, E. Bergeaas Kuutmann, N. Berger, J. Beringer, S. Berlendis, N. R. Bernard, C. Bernius, F. U. Bernlochner, T. Berry, P. Berta, C. Bertella, G. Bertoli, F. Bertolucci, I. A. Bertram, C. Bertsche, D. Bertsche, G. J. Besjes, O. Bessidskaia Bylund, M. Bessner, N. Besson, C. Betancourt, A. Bethani, S. Bethke, A. J. Bevan, R. M. Bianchi, L. Bianchini, M. Bianco, O. Biebel, D. Biedermann, R. Bielski, N. V. Biesuz, M. Biglietti, J. Bilbao De Mendizabal, T. R. V. Billoud, H. Bilokon, M. Bindi, S. Binet, A. Bingul, C. Bini, S. Biondi, T. Bisanz, D. M. Bjergaard, C. W. Black, J. E. Black, K. M. Black, D. Blackburn, R. E. Blair, J. -B. Blanchard, T. Blazek, I. Bloch, C. Blocker, A. Blue, W. Blum, U. Blumenschein, S. Blunier, G. J. Bobbink, V. S. Bobrovnikov, S. S. Bocchetta, A. Bocci, C. Bock, M. Boehler, D. Boerner, J. A. Bogaerts, D. Bogavac, A. G. Bogdanchikov, C. Bohm, V. Boisvert, P. Bokan, T. Bold, A. S. Boldyrev, M. Bomben, M. Bona, M. Boonekamp, A. Borisov, G. Borissov, J. Bortfeldt, D. Bortoletto, V. Bortolotto, K. Bos, D. Boscherini, M. Bosman, J. D. Bossio Sola, J. Boudreau, J. Bouffard, E. V. Bouhova-Thacker, D. Boumediene, C. Bourdarios, S. K. Boutle, A. Boveia, J. Boyd, I. R. Boyko, J. Bracinik, A. Brandt, G. Brandt, O. Brandt, U. Bratzler, B. Brau, J. E. Brau, W. D. Breaden Madden, K. Brendlinger, A. J. Brennan, L. Brenner, R. Brenner, S. Bressler, T. M. Bristow, D. Britton, D. Britzger, F. M. Brochu, I. Brock, R. Brock, G. Brooijmans, T. Brooks, W. K. Brooks, J. Brosamer, E. Brost, J. H Broughton, P. A. Bruckman de Renstrom, D. Bruncko, R. Bruneliere, A. Bruni, G. Bruni, L. S. Bruni, BH Brunt, M. Bruschi, N. Bruscino, P. Bryant, L. Bryngemark, T. Buanes, Q. Buat, P. Buchholz, A. G. Buckley, I. A. Budagov, F. Buehrer, M. K. Bugge, O. Bulekov, D. Bullock, H. Burckhart, S. Burdin, C. D. Burgard, B. Burghgrave, K. Burka, S. Burke, I. Burmeister, J. T. P. Burr, E. Busato, D. Büscher, V. Büscher, P. Bussey, J. M. Butler, C. M. Buttar, J. M. Butterworth, P. Butti, W. Buttinger, A. Buzatu, A. R. Buzykaev, S. Cabrera Urbán, D. Caforio, V. M. Cairo, O. Cakir, N. Calace, P. Calafiura, A. Calandri, G. Calderini, P. Calfayan, G. Callea, L. P. Caloba, S. Calvente Lopez, D. Calvet, S. Calvet, T. P. Calvet, R. Camacho Toro, S. Camarda, P. Camarri, D. Cameron, R. Caminal Armadans, C. Camincher, S. Campana, M. Campanelli, A. Camplani, A. Campoverde, V. Canale, A. Canepa, M. Cano Bret, J. Cantero, T. Cao, M. D. M. Capeans Garrido, I. Caprini, M. Caprini, M. Capua, R. M. Carbone, R. Cardarelli, F. Cardillo, I. Carli, T. Carli, G. Carlino, L. Carminati, S. Caron, E. Carquin, G. D. Carrillo-Montoya, J. R. Carter, J. Carvalho, D. Casadei, M. P. Casado, M. Casolino, D. W. Casper, E. Castaneda-Miranda, R. Castelijn, A. Castelli, V. Castillo Gimenez, N. F. Castro, A. Catinaccio, J. R. Catmore, A. Cattai, J. Caudron, V. Cavaliere, E. Cavallaro, D. Cavalli, M. Cavalli-Sforza, V. Cavasinni, F. Ceradini, L. Cerda Alberich, B. C. Cerio, A. S. Cerqueira, A. Cerri, L. Cerrito, F. Cerutti, M. Cerv, A. Cervelli, S. A. Cetin, A. Chafaq, D. Chakraborty, S. K. Chan, Y. L. Chan, P. Chang, J. D. Chapman, D. G. Charlton, A. Chatterjee, C. C. Chau, C. A. Chavez Barajas, S. Che, S. Cheatham, A. Chegwidden, S. Chekanov, S. V. Chekulaev, G. A. Chelkov, M. A. Chelstowska, C. Chen, H. Chen, K. Chen, S. Chen, S. Chen, X. Chen, Y. Chen, H. C. Cheng, H. J Cheng, Y. Cheng, A. Cheplakov, E. Cheremushkina, R. Cherkaoui El Moursli, V. Chernyatin, E. Cheu, L. Chevalier, V. Chiarella, G. Chiarelli, G. Chiodini, A. S. Chisholm, A. Chitan, M. V. Chizhov, K. Choi, A. R. Chomont, S. Chouridou, B. K. B. Chow, V. Christodoulou, D. Chromek-Burckhart, J. Chudoba, A. J. Chuinard, J. J. Chwastowski, L. Chytka, G. Ciapetti, A. K. Ciftci, D. Cinca, V. Cindro, I. A. Cioara, C. Ciocca, A. Ciocio, F. Cirotto, Z. H. Citron, M. Citterio, M. Ciubancan, A. Clark, B. L. Clark, M. R. Clark, P. J. Clark, R. N. Clarke, C. Clement, Y. Coadou, M. Cobal, A. Coccaro, J. Cochran, L. Colasurdo, B. Cole, A. P. Colijn, J. Collot, T. Colombo, G. Compostella, P. Conde Muiño, E. Coniavitis, S. H. Connell, I. A. Connelly, V. Consorti, S. Constantinescu, G. Conti, F. Conventi, M. Cooke, B. D. Cooper, A. M. Cooper-Sarkar, K. J. R. Cormier, T. Cornelissen, M. Corradi, F. Corriveau, A. Cortes-Gonzalez, G. Cortiana, G. Costa, M. J. Costa, D. Costanzo, G. Cottin, G. Cowan, B. E. Cox, K. Cranmer, S. J. Crawley, G. Cree, S. Crépé-Renaudin, F. Crescioli, W. A. Cribbs, M. Crispin Ortuzar, M. Cristinziani, V. Croft, G. Crosetti, A. Cueto, T. Cuhadar Donszelmann, J. Cummings, M. Curatolo, J. Cúth, H. Czirr, P. Czodrowski, G. D’amen, S. D’Auria, M. D’Onofrio, M. J. Da Cunha Sargedas De Sousa, C. Da Via, W. Dabrowski, T. Dado, T. Dai, O. Dale, F. Dallaire, C. Dallapiccola, M. Dam, J. R. Dandoy, N. P. Dang, A. C. Daniells, N. S. Dann, M. Danninger, M. Dano Hoffmann, V. Dao, G. Darbo, S. Darmora, J. Dassoulas, A. Dattagupta, W. Davey, C. David, T. Davidek, M. Davies, P. Davison, E. Dawe, I. Dawson, K. De, R. de Asmundis, A. De Benedetti, S. De Castro, S. De Cecco, N. De Groot, P. de Jong, H. De la Torre, F. De Lorenzi, A. De Maria, D. De Pedis, A. De Salvo, U. De Sanctis, A. De Santo, J. B. De Vivie De Regie, W. J. Dearnaley, R. Debbe, C. Debenedetti, D. V. Dedovich, N. Dehghanian, I. Deigaard, M. Del Gaudio, J. Del Peso, T. Del Prete, D. Delgove, F. Deliot, C. M. Delitzsch, A. Dell’Acqua, L. Dell’Asta, M. Dell’Orso, M. Della Pietra, D. della Volpe, M. Delmastro, P. A. Delsart, D. A. DeMarco, S. Demers, M. Demichev, A. Demilly, S. P. Denisov, D. Denysiuk, D. Derendarz, J. E. Derkaoui, F. Derue, P. Dervan, K. Desch, C. Deterre, K. Dette, P. O. Deviveiros, A. Dewhurst, S. Dhaliwal, A. Di Ciaccio, L. Di Ciaccio, W. K. Di Clemente, C. Di Donato, A. Di Girolamo, B. Di Girolamo, B. Di Micco, R. Di Nardo, A. Di Simone, R. Di Sipio, D. Di Valentino, C. Diaconu, M. Diamond, F. A. Dias, M. A. Diaz, E. B. Diehl, J. Dietrich, S. Díez Cornell, A. Dimitrievska, J. Dingfelder, P. Dita, S. Dita, F. Dittus, F. Djama, T. Djobava, J. I. Djuvsland, M. A. B. do Vale, D. Dobos, M. Dobre, C. Doglioni, J. Dolejsi, Z. Dolezal, M. Donadelli, S. Donati, P. Dondero, J. Donini, J. Dopke, A. Doria, M. T. Dova, A. T. Doyle, E. Drechsler, M. Dris, Y. Du, J. Duarte-Campderros, E. Duchovni, G. Duckeck, O. A. Ducu, D. Duda, A. Dudarev, A. Chr. Dudder, E. M. Duffield, L. Duflot, M. Dührssen, M. Dumancic, M. Dunford, H. Duran Yildiz, M. Düren, A. Durglishvili, D. Duschinger, B. Dutta, M. Dyndal, C. Eckardt, K. M. Ecker, R. C. Edgar, N. C. Edwards, T. Eifert, G. Eigen, K. Einsweiler, T. Ekelof, M. El Kacimi, V. Ellajosyula, M. Ellert, S. Elles, F. Ellinghaus, A. A. Elliot, N. Ellis, J. Elmsheuser, M. Elsing, D. Emeliyanov, Y. Enari, O. C. Endner, J. S. Ennis, J. Erdmann, A. Ereditato, G. Ernis, J. Ernst, M. Ernst, S. Errede, E. Ertel, M. Escalier, H. Esch, C. Escobar, B. Esposito, A. I. Etienvre, E. Etzion, H. Evans, A. Ezhilov, M. Ezzi, F. Fabbri, L. Fabbri, G. Facini, R. M. Fakhrutdinov, S. Falciano, R. J. Falla, J. Faltova, Y. Fang, M. Fanti, A. Farbin, A. Farilla, C. Farina, E. M. Farina, T. Farooque, S. Farrell, S. M. Farrington, P. Farthouat, F. Fassi, P. Fassnacht, D. Fassouliotis, M. Faucci Giannelli, A. Favareto, W. J. Fawcett, L. Fayard, O. L. Fedin, W. Fedorko, S. Feigl, L. Feligioni, C. Feng, E. J. Feng, H. Feng, A. B. Fenyuk, L. Feremenga, P. Fernandez Martinez, S. Fernandez Perez, J. Ferrando, A. Ferrari, P. Ferrari, R. Ferrari, D. E. Ferreira de Lima, A. Ferrer, D. Ferrere, C. Ferretti, A. Ferretto Parodi, F. Fiedler, A. Filipčič, M. Filipuzzi, F. Filthaut, M. Fincke-Keeler, K. D. Finelli, M. C. N. Fiolhais, L. Fiorini, A. Firan, A. Fischer, C. Fischer, J. Fischer, W. C. Fisher, N. Flaschel, I. Fleck, P. Fleischmann, G. T. Fletcher, R. R. M. Fletcher, T. Flick, L. R. Flores Castillo, M. J. Flowerdew, G. T. Forcolin, A. Formica, A. Forti, A. G. Foster, D. Fournier, H. Fox, S. Fracchia, P. Francavilla, M. Franchini, D. Francis, L. Franconi, M. Franklin, M. Frate, M. Fraternali, D. Freeborn, S. M. Fressard-Batraneanu, F. Friedrich, D. Froidevaux, J. A. Frost, C. Fukunaga, E. Fullana Torregrosa, T. Fusayasu, J. Fuster, C. Gabaldon, O. Gabizon, A. Gabrielli, A. Gabrielli, G. P. Gach, S. Gadatsch, S. Gadomski, G. Gagliardi, L. G. Gagnon, P. Gagnon, C. Galea, B. Galhardo, E. J. Gallas, B. J. Gallop, P. Gallus, G. Galster, K. K. Gan, J. Gao, Y. Gao, Y. S. Gao, F. M. Garay Walls, C. García, J. E. García Navarro, M. Garcia-Sciveres, R. W. Gardner, N. Garelli, V. Garonne, A. Gascon Bravo, K. Gasnikova, C. Gatti, A. Gaudiello, G. Gaudio, L. Gauthier, I. L. Gavrilenko, C. Gay, G. Gaycken, E. N. Gazis, Z. Gecse, C. N. P. Gee, Ch. Geich-Gimbel, M. Geisen, M. P. Geisler, K. Gellerstedt, C. Gemme, M. H. Genest, C. Geng, S. Gentile, C. Gentsos, S. George, D. Gerbaudo, A. Gershon, S. Ghasemi, M. Ghneimat, B. Giacobbe, S. Giagu, P. Giannetti, B. Gibbard, S. M. Gibson, M. Gignac, M. Gilchriese, T. P. S. Gillam, D. Gillberg, G. Gilles, D. M. Gingrich, N. Giokaris, M. P. Giordani, F. M. Giorgi, F. M. Giorgi, P. F. Giraud, P. Giromini, D. Giugni, F. Giuli, C. Giuliani, M. Giulini, B. K. Gjelsten, S. Gkaitatzis, I. Gkialas, E. L. Gkougkousis, L. K. Gladilin, C. Glasman, J. Glatzer, P. C. F. Glaysher, A. Glazov, M. Goblirsch-Kolb, J. Godlewski, S. Goldfarb, T. Golling, D. Golubkov, A. Gomes, R. Gonçalo, J. Goncalves Pinto Firmino Da Costa, G. Gonella, L. Gonella, A. Gongadze, S. González de la Hoz, G. Gonzalez Parra, S. Gonzalez-Sevilla, L. Goossens, P. A. Gorbounov, H. A. Gordon, I. Gorelov, B. Gorini, E. Gorini, A. Gorišek, E. Gornicki, A. T. Goshaw, C. Gössling, M. I. Gostkin, C. R. Goudet, D. Goujdami, A. G. Goussiou, N. Govender, E. Gozani, L. Graber, I. Grabowska-Bold, P. O. J. Gradin, P. Grafström, J. Gramling, E. Gramstad, S. Grancagnolo, V. Gratchev, P. M. Gravila, H. M. Gray, E. Graziani, Z. D. Greenwood, C. Grefe, K. Gregersen, I. M. Gregor, P. Grenier, K. Grevtsov, J. Griffiths, A. A. Grillo, K. Grimm, S. Grinstein, Ph. Gris, J. -F. Grivaz, S. Groh, J. P. Grohs, E. Gross, J. Grosse-Knetter, G. C. Grossi, Z. J. Grout, L. Guan, W. Guan, J. Guenther, F. Guescini, D. Guest, O. Gueta, E. Guido, T. Guillemin, S. Guindon, U. Gul, C. Gumpert, J. Guo, Y. Guo, R. Gupta, S. Gupta, G. Gustavino, P. Gutierrez, N. G. Gutierrez Ortiz, C. Gutschow, C. Guyot, C. Gwenlan, C. B. Gwilliam, A. Haas, C. Haber, H. K. Hadavand, N. Haddad, A. Hadef, S. Hageböck, M. Hagihara, Z. Hajduk, H. Hakobyan, M. Haleem, J. Haley, G. Halladjian, G. D. Hallewell, K. Hamacher, P. Hamal, K. Hamano, A. Hamilton, G. N. Hamity, P. G. Hamnett, L. Han, K. Hanagaki, K. Hanawa, M. Hance, B. Haney, P. Hanke, R. Hanna, J. B. Hansen, J. D. Hansen, M. C. Hansen, P. H. Hansen, K. Hara, A. S. Hard, T. Harenberg, F. Hariri, S. Harkusha, R. D. Harrington, P. F. Harrison, F. Hartjes, N. M. Hartmann, M. Hasegawa, Y. Hasegawa, A. Hasib, S. Hassani, S. Haug, R. Hauser, L. Hauswald, M. Havranek, C. M. Hawkes, R. J. Hawkings, D. Hayakawa, D. Hayden, C. P. Hays, J. M. Hays, H. S. Hayward, S. J. Haywood, S. J. Head, T. Heck, V. Hedberg, L. Heelan, S. Heim, T. Heim, B. Heinemann, J. J. Heinrich, L. Heinrich, C. Heinz, J. Hejbal, L. Helary, S. Hellman, C. Helsens, J. Henderson, R. C. W. Henderson, Y. Heng, S. Henkelmann, A. M. Henriques Correia, S. Henrot-Versille, G. H. Herbert, H. Herde, V. Herget, Y. Hernández Jiménez, G. Herten, R. Hertenberger, L. Hervas, G. G. Hesketh, N. P. Hessey, J. W. Hetherly, R. Hickling, E. Higón-Rodriguez, E. Hill, J. C. Hill, K. H. Hiller, S. J. Hillier, I. Hinchliffe, E. Hines, R. R. Hinman, M. Hirose, D. Hirschbuehl, J. Hobbs, N. Hod, M. C. Hodgkinson, P. Hodgson, A. Hoecker, M. R. Hoeferkamp, F. Hoenig, D. Hohn, T. R. Holmes, M. Homann, T. Honda, T. M. Hong, B. H. Hooberman, W. H. Hopkins, Y. Horii, A. J. Horton, J-Y. Hostachy, S. Hou, A. Hoummada, J. Howarth, J. Hoya, M. Hrabovsky, I. Hristova, J. Hrivnac, T. Hryn’ova, A. Hrynevich, C. Hsu, P. J. Hsu, S. -C. Hsu, Q. Hu, S. Hu, Y. Huang, Z. Hubacek, F. Hubaut, F. Huegging, T. B. Huffman, E. W. Hughes, G. Hughes, M. Huhtinen, P. Huo, N. Huseynov, J. Huston, J. Huth, G. Iacobucci, G. Iakovidis, I. Ibragimov, L. Iconomidou-Fayard, E. Ideal, Z. Idrissi, P. Iengo, O. Igonkina, T. Iizawa, Y. Ikegami, M. Ikeno, Y. Ilchenko, D. Iliadis, N. Ilic, T. Ince, G. Introzzi, P. Ioannou, M. Iodice, K. Iordanidou, V. Ippolito, N. Ishijima, M. Ishino, M. Ishitsuka, R. Ishmukhametov, C. Issever, S. Istin, F. Ito, J. M. Iturbe Ponce, R. Iuppa, W. Iwanski, H. Iwasaki, J. M. Izen, V. Izzo, S. Jabbar, B. Jackson, P. Jackson, V. Jain, K. B. Jakobi, K. Jakobs, S. Jakobsen, T. Jakoubek, D. O. Jamin, D. K. Jana, R. Jansky, J. Janssen, M. Janus, G. Jarlskog, N. Javadov, T. Javůrek, F. Jeanneau, L. Jeanty, G. -Y. Jeng, D. Jennens, P. Jenni, C. Jeske, S. Jézéquel, H. Ji, J. Jia, H. Jiang, Y. Jiang, S. Jiggins, J. Jimenez Pena, S. Jin, A. Jinaru, O. Jinnouchi, H. Jivan, P. Johansson, K. A. Johns, W. J. Johnson, K. Jon-And, G. Jones, R. W. L. Jones, S. Jones, T. J. Jones, J. Jongmanns, P. M. Jorge, J. Jovicevic, X. Ju, A. Juste Rozas, M. K. Köhler, A. Kaczmarska, M. Kado, H. Kagan, M. Kagan, S. J. Kahn, T. Kaji, E. Kajomovitz, C. W. Kalderon, A. Kaluza, S. Kama, A. Kamenshchikov, N. Kanaya, S. Kaneti, L. Kanjir, V. A. Kantserov, J. Kanzaki, B. Kaplan, L. S. Kaplan, A. Kapliy, D. Kar, K. Karakostas, A. Karamaoun, N. Karastathis, M. J. Kareem, E. Karentzos, M. Karnevskiy, S. N. Karpov, Z. M. Karpova, K. Karthik, V. Kartvelishvili, A. N. Karyukhin, K. Kasahara, L. Kashif, R. D. Kass, A. Kastanas, Y. Kataoka, C. Kato, A. Katre, J. Katzy, K. Kawagoe, T. Kawamoto, G. Kawamura, V. F. Kazanin, R. Keeler, R. Kehoe, J. S. Keller, J. J. Kempster, K Kentaro, H. Keoshkerian, O. Kepka, B. P. Kerševan, S. Kersten, R. A. Keyes, M. Khader, F. Khalil-zada, A. Khanov, A. G. Kharlamov, T. Kharlamova, T. J. Khoo, V. Khovanskiy, E. Khramov, J. Khubua, S. Kido, C. R. Kilby, H. Y. Kim, S. H. Kim, Y. K. Kim, N. Kimura, O. M. Kind, B. T. King, M. King, J. Kirk, A. E. Kiryunin, T. Kishimoto, D. Kisielewska, F. Kiss, K. Kiuchi, O. Kivernyk, E. Kladiva, M. H. Klein, M. Klein, U. Klein, K. Kleinknecht, P. Klimek, A. Klimentov, R. Klingenberg, J. A. Klinger, T. Klioutchnikova, E. -E. Kluge, P. Kluit, S. Kluth, J. Knapik, E. Kneringer, E. B. F. G. Knoops, A. Knue, A. Kobayashi, D. Kobayashi, T. Kobayashi, M. Kobel, M. Kocian, P. Kodys, N. M. Koehler, T. Koffas, E. Koffeman, T. Koi, H. Kolanoski, M. Kolb, I. Koletsou, A. A. Komar, Y. Komori, T. Kondo, N. Kondrashova, K. Köneke, A. C. König, T. Kono, R. Konoplich, N. Konstantinidis, R. Kopeliansky, S. Koperny, L. Köpke, A. K. Kopp, K. Korcyl, K. Kordas, A. Korn, A. A. Korol, I. Korolkov, E. V. Korolkova, O. Kortner, S. Kortner, T. Kosek, V. V. Kostyukhin, A. Kotwal, A. Kourkoumeli-Charalampidi, C. Kourkoumelis, V. Kouskoura, A. B. Kowalewska, R. Kowalewski, T. Z. Kowalski, C. Kozakai, W. Kozanecki, A. S. Kozhin, V. A. Kramarenko, G. Kramberger, D. Krasnopevtsev, M. W. Krasny, A. Krasznahorkay, A. Kravchenko, M. Kretz, J. Kretzschmar, K. Kreutzfeldt, P. Krieger, K. Krizka, K. Kroeninger, H. Kroha, J. Kroll, J. Kroseberg, J. Krstic, U. Kruchonak, H. Krüger, N. Krumnack, M. C. Kruse, M. Kruskal, T. Kubota, H. Kucuk, S. Kuday, J. T. Kuechler, S. Kuehn, A. Kugel, F. Kuger, A. Kuhl, T. Kuhl, V. Kukhtin, R. Kukla, Y. Kulchitsky, S. Kuleshov, M. Kuna, T. Kunigo, A. Kupco, H. Kurashige, Y. A. Kurochkin, V. Kus, E. S. Kuwertz, M. Kuze, J. Kvita, T. Kwan, D. Kyriazopoulos, A. La Rosa, J. L. La Rosa Navarro, L. La Rotonda, C. Lacasta, F. Lacava, J. Lacey, H. Lacker, D. Lacour, V. R. Lacuesta, E. Ladygin, R. Lafaye, B. Laforge, T. Lagouri, S. Lai, S. Lammers, W. Lampl, E. Lançon, U. Landgraf, M. P. J. Landon, M. C. Lanfermann, V. S. Lang, J. C. Lange, A. J. Lankford, F. Lanni, K. Lantzsch, A. Lanza, S. Laplace, C. Lapoire, J. F. Laporte, T. Lari, F. Lasagni Manghi, M. Lassnig, P. Laurelli, W. Lavrijsen, A. T. Law, P. Laycock, T. Lazovich, M. Lazzaroni, B. Le, O. Le Dortz, E. Le Guirriec, E. P. Le Quilleuc, M. LeBlanc, T. LeCompte, F. Ledroit-Guillon, C. A. Lee, S. C. Lee, L. Lee, B. Lefebvre, G. Lefebvre, M. Lefebvre, F. Legger, C. Leggett, A. Lehan, G. Lehmann Miotto, X. Lei, W. A. Leight, A. Leisos, A. G. Leister, M. A. L. Leite, R. Leitner, D. Lellouch, B. Lemmer, K. J. C. Leney, T. Lenz, B. Lenzi, R. Leone, S. Leone, C. Leonidopoulos, S. Leontsinis, G. Lerner, C. Leroy, A. A. J. Lesage, C. G. Lester, M. Levchenko, J. Levêque, D. Levin, L. J. Levinson, M. Levy, D. Lewis, A. M. Leyko, M. Leyton, B. Li, C. Li, H. Li, H. L. Li, L. Li, L. Li, Q. Li, S. Li, X. Li, Y. Li, Z. Liang, B. Liberti, A. Liblong, P. Lichard, K. Lie, J. Liebal, W. Liebig, A. Limosani, S. C. Lin, T. H. Lin, B. E. Lindquist, A. E. Lionti, E. Lipeles, A. Lipniacka, M. Lisovyi, T. M. Liss, A. Lister, A. M. Litke, B. Liu, D. Liu, H. Liu, H. Liu, J. Liu, J. B. Liu, K. Liu, L. Liu, M. Liu, M. Liu, Y. L. Liu, Y. Liu, M. Livan, A. Lleres, J. Llorente Merino, S. L. Lloyd, F. Lo Sterzo, E. M. Lobodzinska, P. Loch, W. S. Lockman, F. K. Loebinger, A. E. Loevschall-Jensen, K. M. Loew, A. Loginov, T. Lohse, K. Lohwasser, M. Lokajicek, B. A. Long, J. D. Long, R. E. Long, L. Longo, K. A. Looper, J. A. López, D. Lopez Mateos, B. Lopez Paredes, I. Lopez Paz, A. Lopez Solis, J. Lorenz, N. Lorenzo Martinez, M. Losada, P. J. Lösel, X. Lou, A. Lounis, J. Love, P. A. Love, H. Lu, N. Lu, H. J. Lubatti, C. Luci, A. Lucotte, C. Luedtke, F. Luehring, W. Lukas, L. Luminari, O. Lundberg, B. Lund-Jensen, P. M. Luzi, D. Lynn, R. Lysak, E. Lytken, V. Lyubushkin, H. Ma, L. L. Ma, Y. Ma, G. Maccarrone, A. Macchiolo, C. M. Macdonald, B. Maček, J. Machado Miguens, D. Madaffari, R. Madar, H. J. Maddocks, W. F. Mader, A. Madsen, J. Maeda, S. Maeland, T. Maeno, A. Maevskiy, E. Magradze, J. Mahlstedt, C. Maiani, C. Maidantchik, A. A. Maier, T. Maier, A. Maio, S. Majewski, Y. Makida, N. Makovec, B. Malaescu, Pa. Malecki, V. P. Maleev, F. Malek, U. Mallik, D. Malon, C. Malone, C. Malone, S. Maltezos, S. Malyukov, J. Mamuzic, G. Mancini, L. Mandelli, I. Mandić, J. Maneira, L. Manhaes de Andrade Filho, J. Manjarres Ramos, A. Mann, A. Manousos, B. Mansoulie, J. D. Mansour, R. Mantifel, M. Mantoani, S. Manzoni, L. Mapelli, G. Marceca, L. March, G. Marchiori, M. Marcisovsky, M. Marjanovic, D. E. Marley, F. Marroquim, S. P. Marsden, Z. Marshall, S. Marti-Garcia, B. Martin, T. A. Martin, V. J. Martin, B. Martin dit Latour, M. Martinez, V. I. Martinez Outschoorn, S. Martin-Haugh, V. S. Martoiu, A. C. Martyniuk, A. Marzin, L. Masetti, T. Mashimo, R. Mashinistov, J. Masik, A. L. Maslennikov, I. Massa, L. Massa, P. Mastrandrea, A. Mastroberardino, T. Masubuchi, P. Mättig, J. Mattmann, J. Maurer, S. J. Maxfield, D. A. Maximov, R. Mazini, S. M. Mazza, N. C. Mc Fadden, G. Mc Goldrick, S. P. Mc Kee, A. McCarn, R. L. McCarthy, T. G. McCarthy, L. I. McClymont, E. F. McDonald, J. A. Mcfayden, G. Mchedlidze, S. J. McMahon, R. A. McPherson, M. Medinnis, S. Meehan, S. Mehlhase, A. Mehta, K. Meier, C. Meineck, B. Meirose, D. Melini, B. R. Mellado Garcia, M. Melo, F. Meloni, A. Mengarelli, S. Menke, E. Meoni, S. Mergelmeyer, P. Mermod, L. Merola, C. Meroni, F. S. Merritt, A. Messina, J. Metcalfe, A. S. Mete, C. Meyer, C. Meyer, J-P. Meyer, J. Meyer, H. Meyer Zu Theenhausen, F. Miano, R. P. Middleton, S. Miglioranzi, L. Mijović, G. Mikenberg, M. Mikestikova, M. Mikuž, M. Milesi, A. Milic, D. W. Miller, C. Mills, A. Milov, D. A. Milstead, A. A. Minaenko, Y. Minami, I. A. Minashvili, A. I. Mincer, B. Mindur, M. Mineev, Y. Minegishi, Y. Ming, L. M. Mir, K. P. Mistry, T. Mitani, J. Mitrevski, V. A. Mitsou, A. Miucci, P. S. Miyagawa, J. U. Mjörnmark, M. Mlynarikova, T. Moa, K. Mochizuki, S. Mohapatra, S. Molander, R. Moles-Valls, R. Monden, M. C. Mondragon, K. Mönig, J. Monk, E. Monnier, A. Montalbano, J. Montejo Berlingen, F. Monticelli, S. Monzani, R. W. Moore, N. Morange, D. Moreno, M. Moreno Llácer, P. Morettini, S. Morgenstern, D. Mori, T. Mori, M. Morii, M. Morinaga, V. Morisbak, S. Moritz, A. K. Morley, G. Mornacchi, J. D. Morris, S. S. Mortensen, L. Morvaj, M. Mosidze, J. Moss, K. Motohashi, R. Mount, E. Mountricha, E. J. W. Moyse, S. Muanza, R. D. Mudd, F. Mueller, J. Mueller, R. S. P. Mueller, T. Mueller, D. Muenstermann, P. Mullen, G. A. Mullier, F. J. Munoz Sanchez, J. A. Murillo Quijada, W. J. Murray, H. Musheghyan, M. Muškinja, A. G. Myagkov, M. Myska, B. P. Nachman, O. Nackenhorst, K. Nagai, R. Nagai, K. Nagano, Y. Nagasaka, K. Nagata, M. Nagel, E. Nagy, A. M. Nairz, Y. Nakahama, K. Nakamura, T. Nakamura, I. Nakano, H. Namasivayam, R. F. Naranjo Garcia, R. Narayan, D. I. Narrias Villar, I. Naryshkin, T. Naumann, G. Navarro, R. Nayyar, H. A. Neal, P. Yu. Nechaeva, T. J. Neep, A. Negri, M. Negrini, S. Nektarijevic, C. Nellist, A. Nelson, S. Nemecek, P. Nemethy, A. A. Nepomuceno, M. Nessi, M. S. Neubauer, M. Neumann, R. M. Neves, P. Nevski, P. R. Newman, D. H. Nguyen, T. Nguyen Manh, R. B. Nickerson, R. Nicolaidou, J. Nielsen, A. Nikiforov, V. Nikolaenko, I. Nikolic-Audit, K. Nikolopoulos, J. K. Nilsen, P. Nilsson, Y. Ninomiya, A. Nisati, R. Nisius, T. Nobe, M. Nomachi, I. Nomidis, T. Nooney, S. Norberg, M. Nordberg, N. Norjoharuddeen, O. Novgorodova, S. Nowak, M. Nozaki, L. Nozka, K. Ntekas, E. Nurse, F. Nuti, F. O’grady, D. C. O’Neil, A. A. O’Rourke, V. O’Shea, F. G. Oakham, H. Oberlack, T. Obermann, J. Ocariz, A. Ochi, I. Ochoa, J. P. Ochoa-Ricoux, S. Oda, S. Odaka, H. Ogren, A. Oh, S. H. Oh, C. C. Ohm, H. Ohman, H. Oide, H. Okawa, Y. Okumura, T. Okuyama, A. Olariu, L. F. Oleiro Seabra, S. A. Olivares Pino, D. Damazio, A. Olszewski, J. Olszowska, A. Onofre, K. Onogi, P. U. E. Onyisi, M. J. Oreglia, Y. Oren, D. Orestano, N. Orlando, R. S. Orr, B. Osculati, R. Ospanov, G. Otero y Garzon, H. Otono, M. Ouchrif, F. Ould-Saada, A. Ouraou, K. P. Oussoren, Q. Ouyang, M. Owen, R. E. Owen, V. E. Ozcan, N. Ozturk, K. Pachal, A. Pacheco Pages, L. Pacheco Rodriguez, C. Padilla Aranda, M. Pagáčová, S. Pagan Griso, M. Paganini, F. Paige, P. Pais, K. Pajchel, G. Palacino, S. Palazzo, S. Palestini, M. Palka, D. Pallin, E. St. Panagiotopoulou, C. E. Pandini, J. G. Panduro Vazquez, P. Pani, S. Panitkin, D. Pantea, L. Paolozzi, Th. D. Papadopoulou, K. Papageorgiou, A. Paramonov, D. Paredes Hernandez, A. J. Parker, M. A. Parker, K. A. Parker, F. Parodi, J. A. Parsons, U. Parzefall, V. R. Pascuzzi, E. Pasqualucci, S. Passaggio, Fr. Pastore, G. Pásztor, S. Pataraia, J. R. Pater, T. Pauly, J. Pearce, B. Pearson, L. E. Pedersen, M. Pedersen, S. Pedraza Lopez, R. Pedro, S. V. Peleganchuk, O. Penc, C. Peng, H. Peng, J. Penwell, B. S. Peralva, M. M. Perego, D. V. Perepelitsa, E. Perez Codina, L. Perini, H. Pernegger, S. Perrella, R. Peschke, V. D. Peshekhonov, K. Peters, R. F. Y. Peters, B. A. Petersen, T. C. Petersen, E. Petit, A. Petridis, C. Petridou, P. Petroff, E. Petrolo, M. Petrov, F. Petrucci, N. E. Pettersson, A. Peyaud, R. Pezoa, P. W. Phillips, G. Piacquadio, E. Pianori, A. Picazio, E. Piccaro, M. Piccinini, M. A. Pickering, R. Piegaia, J. E. Pilcher, A. D. Pilkington, A. W. J. Pin, M. Pinamonti, J. L. Pinfold, A. Pingel, S. Pires, H. Pirumov, M. Pitt, L. Plazak, M. -A. Pleier, V. Pleskot, E. Plotnikova, P. Plucinski, D. Pluth, R. Poettgen, L. Poggioli, D. Pohl, G. Polesello, A. Poley, A. Policicchio, R. Polifka, A. Polini, C. S. Pollard, V. Polychronakos, K. Pommès, L. Pontecorvo, B. G. Pope, G. A. Popeneciu, A. Poppleton, S. Pospisil, K. Potamianos, I. N. Potrap, C. J. Potter, C. T. Potter, G. Poulard, J. Poveda, V. Pozdnyakov, M. E. Pozo Astigarraga, P. Pralavorio, A. Pranko, S. Prell, D. Price, L. E. Price, M. Primavera, S. Prince, K. Prokofiev, F. Prokoshin, S. Protopopescu, J. Proudfoot, M. Przybycien, D. Puddu, M. Purohit, P. Puzo, J. Qian, G. Qin, Y. Qin, A. Quadt, W. B. Quayle, M. Queitsch-Maitland, D. Quilty, S. Raddum, V. Radeka, V. Radescu, S. K. Radhakrishnan, P. Radloff, P. Rados, F. Ragusa, G. Rahal, J. A. Raine, S. Rajagopalan, M. Rammensee, C. Rangel-Smith, M. G. Ratti, F. Rauscher, S. Rave, T. Ravenscroft, I. Ravinovich, M. Raymond, A. L. Read, N. P. Readioff, M. Reale, D. M. Rebuzzi, A. Redelbach, G. Redlinger, R. Reece, R. G. Reed, K. Reeves, L. Rehnisch, J. Reichert, A. Reiss, C. Rembser, H. Ren, M. Rescigno, S. Resconi, O. L. Rezanova, P. Reznicek, R. Rezvani, R. Richter, S. Richter, E. Richter-Was, O. Ricken, M. Ridel, P. Rieck, C. J. Riegel, J. Rieger, O. Rifki, M. Rijssenbeek, A. Rimoldi, M. Rimoldi, L. Rinaldi, B. Ristić, E. Ritsch, I. Riu, F. Rizatdinova, E. Rizvi, C. Rizzi, S. H. Robertson, A. Robichaud-Veronneau, D. Robinson, J. E. M. Robinson, A. Robson, C. Roda, Y. Rodina, A. Rodriguez Perez, D. Rodriguez Rodriguez, S. Roe, C. S. Rogan, O. Røhne, A. Romaniouk, M. Romano, S. M. Romano Saez, E. Romero Adam, N. Rompotis, M. Ronzani, L. Roos, E. Ros, S. Rosati, K. Rosbach, P. Rose, N. -A. Rosien, V. Rossetti, E. Rossi, L. P. Rossi, J. H. N. Rosten, R. Rosten, M. Rotaru, I. Roth, J. Rothberg, D. Rousseau, A. Rozanov, Y. Rozen, X. Ruan, F. Rubbo, M. S. Rudolph, F. Rühr, A. Ruiz-Martinez, Z. Rurikova, N. A. Rusakovich, A. Ruschke, H. L. Russell, J. P. Rutherfoord, N. Ruthmann, Y. F. Ryabov, M. Rybar, G. Rybkin, S. Ryu, A. Ryzhov, G. F. Rzehorz, A. F. Saavedra, G. Sabato, S. Sacerdoti, H. F-W. Sadrozinski, R. Sadykov, F. Safai Tehrani, P. Saha, M. Sahinsoy, M. Saimpert, T. Saito, H. Sakamoto, Y. Sakurai, G. Salamanna, A. Salamon, J. E. Salazar Loyola, D. Salek, P. H. Sales De Bruin, D. Salihagic, A. Salnikov, J. Salt, D. Salvatore, F. Salvatore, A. Salvucci, A. Salzburger, D. Sammel, D. Sampsonidis, A. Sanchez, J. Sánchez, V. Sanchez Martinez, H. Sandaker, R. L. Sandbach, H. G. Sander, M. Sandhoff, C. Sandoval, D. P. C. Sankey, M. Sannino, A. Sansoni, C. Santoni, R. Santonico, H. Santos, I. Santoyo Castillo, K. Sapp, A. Sapronov, J. G. Saraiva, B. Sarrazin, O. Sasaki, K. Sato, E. Sauvan, G. Savage, P. Savard, N. Savic, C. Sawyer, L. Sawyer, J. Saxon, C. Sbarra, A. Sbrizzi, T. Scanlon, D. A. Scannicchio, M. Scarcella, V. Scarfone, J. Schaarschmidt, P. Schacht, B. M. Schachtner, D. Schaefer, L. Schaefer, R. Schaefer, J. Schaeffer, S. Schaepe, S. Schaetzel, U. Schäfer, A. C. Schaffer, D. Schaile, R. D. Schamberger, V. Scharf, V. A. Schegelsky, D. Scheirich, M. Schernau, C. Schiavi, S. Schier, C. Schillo, M. Schioppa, S. Schlenker, K. R. Schmidt-Sommerfeld, K. Schmieden, C. Schmitt, S. Schmitt, S. Schmitz, B. Schneider, U. Schnoor, L. Schoeffel, A. Schoening, B. D. Schoenrock, E. Schopf, M. Schott, J. F. P. Schouwenberg, J. Schovancova, S. Schramm, M. Schreyer, N. Schuh, A. Schulte, M. J. Schultens, H.-C. Schultz-Coulon, H. Schulz, M. Schumacher, B. A. Schumm, Ph. Schune, A. Schwartzman, T. A. Schwarz, H. Schweiger, Ph. Schwemling, R. Schwienhorst, J. Schwindling, T. Schwindt, G. Sciolla, F. Scuri, F. Scutti, J. Searcy, P. Seema, S. C. Seidel, A. Seiden, F. Seifert, J. M. Seixas, G. Sekhniaidze, K. Sekhon, S. J. Sekula, D. M. Seliverstov, N. Semprini-Cesari, C. Serfon, L. Serin, L. Serkin, M. Sessa, R. Seuster, H. Severini, T. Sfiligoj, F. Sforza, A. Sfyrla, E. Shabalina, N. W. Shaikh, L. Y. Shan, R. Shang, J. T. Shank, M. Shapiro, P. B. Shatalov, K. Shaw, S. M. Shaw, A. Shcherbakova, C. Y. Shehu, P. Sherwood, L. Shi, S. Shimizu, C. O. Shimmin, M. Shimojima, S. Shirabe, M. Shiyakova, A. Shmeleva, D. Shoaleh Saadi, M. J. Shochet, S. Shojaii, D. R. Shope, S. Shrestha, E. Shulga, M. A. Shupe, P. Sicho, A. M. Sickles, P. E. Sidebo, O. Sidiropoulou, D. Sidorov, A. Sidoti, F. Siegert, Dj. Sijacki, J. Silva, S. B. Silverstein, V. Simak, Lj. Simic, S. Simion, E. Simioni, B. Simmons, D. Simon, M. Simon, P. Sinervo, N. B. Sinev, M. Sioli, G. Siragusa, S. Yu. Sivoklokov, J. Sjölin, M. B. Skinner, H. P. Skottowe, P. Skubic, M. Slater, T. Slavicek, M. Slawinska, K. Sliwa, R. Slovak, V. Smakhtin, B. H. Smart, L. Smestad, J. Smiesko, S. Yu. Smirnov, Y. Smirnov, L. N. Smirnova, O. Smirnova, M. N. K. Smith, R. W. Smith, M. Smizanska, K. Smolek, A. A. Snesarev, I. M. Snyder, S. Snyder, R. Sobie, F. Socher, A. Soffer, D. A. Soh, G. Sokhrannyi, C. A. Solans Sanchez, M. Solar, E. Yu. Soldatov, U. Soldevila, A. A. Solodkov, A. Soloshenko, O. V. Solovyanov, V. Solovyev, P. Sommer, H. Son, H. Y. Song, A. Sood, A. Sopczak, V. Sopko, V. Sorin, D. Sosa, C. L. Sotiropoulou, R. Soualah, A. M. Soukharev, D. South, B. C. Sowden, S. Spagnolo, M. Spalla, M. Spangenberg, F. Spanò, D. Sperlich, F. Spettel, R. Spighi, G. Spigo, L. A. Spiller, M. Spousta, R. D. St. Denis, A. Stabile, R. Stamen, S. Stamm, E. Stanecka, R. W. Stanek, C. Stanescu, M. Stanescu-Bellu, M. M. Stanitzki, S. Stapnes, E. A. Starchenko, G. H. Stark, J. Stark, P. Staroba, P. Starovoitov, S. Stärz, R. Staszewski, P. Steinberg, B. Stelzer, H. J. Stelzer, O. Stelzer-Chilton, H. Stenzel, G. A. Stewart, J. A. Stillings, M. C. Stockton, M. Stoebe, G. Stoicea, P. Stolte, S. Stonjek, A. R. Stradling, A. Straessner, M. E. Stramaglia, J. Strandberg, S. Strandberg, A. Strandlie, M. Strauss, P. Strizenec, R. Ströhmer, D. M. Strom, R. Stroynowski, A. Strubig, S. A. Stucci, B. Stugu, N. A. Styles, D. Su, J. Su, S. Suchek, Y. Sugaya, M. Suk, V. V. Sulin, S. Sultansoy, T. Sumida, S. Sun, X. Sun, J. E. Sundermann, K. Suruliz, G. Susinno, M. R. Sutton, S. Suzuki, M. Svatos, M. Swiatlowski, I. Sykora, T. Sykora, D. Ta, C. Taccini, K. Tackmann, J. Taenzer, A. Taffard, R. Tafirout, N. Taiblum, H. Takai, R. Takashima, T. Takeshita, Y. Takubo, M. Talby, A. A. Talyshev, K. G. Tan, J. Tanaka, M. Tanaka, R. Tanaka, S. Tanaka, R. Tanioka, B. B. Tannenwald, S. Tapia Araya, S. Tapprogge, S. Tarem, G. F. Tartarelli, P. Tas, M. Tasevsky, T. Tashiro, E. Tassi, A. Tavares Delgado, Y. Tayalati, A. C. Taylor, G. N. Taylor, P. T. E. Taylor, W. Taylor, F. A. Teischinger, P. Teixeira-Dias, K. K. Temming, D. Temple, H. Ten Kate, P. K. Teng, J. J. Teoh, F. Tepel, S. Terada, K. Terashi, J. Terron, S. Terzo, M. Testa, R. J. Teuscher, T. Theveneaux-Pelzer, J. P. Thomas, J. Thomas-Wilsker, E. N. Thompson, P. D. Thompson, A. S. Thompson, L. A. Thomsen, E. Thomson, M. Thomson, M. J. Tibbetts, R. E. Ticse Torres, V. O. Tikhomirov, Yu. A. Tikhonov, S. Timoshenko, P. Tipton, S. Tisserant, K. Todome, T. Todorov, S. Todorova-Nova, J. Tojo, S. Tokár, K. Tokushuku, E. Tolley, L. Tomlinson, M. Tomoto, L. Tompkins, K. Toms, B. Tong, P. Tornambe, E. Torrence, H. Torres, E. Torró Pastor, J. Toth, F. Touchard, D. R. Tovey, T. Trefzger, A. Tricoli, I. M. Trigger, S. Trincaz-Duvoid, M. F. Tripiana, W. Trischuk, B. Trocmé, A. Trofymov, C. Troncon, M. Trottier-McDonald, M. Trovatelli, L. Truong, M. Trzebinski, A. Trzupek, J. C.-L. Tseng, P. V. Tsiareshka, G. Tsipolitis, N. Tsirintanis, S. Tsiskaridze, V. Tsiskaridze, E. G. Tskhadadze, K. M. Tsui, I. I. Tsukerman, V. Tsulaia, S. Tsuno, D. Tsybychev, Y. Tu, A. Tudorache, V. Tudorache, A. N. Tuna, S. A. Tupputi, S. Turchikhin, D. Turecek, D. Turgeman, R. Turra, P. M. Tuts, M. Tyndel, G. Ucchielli, I. Ueda, M. Ughetto, F. Ukegawa, G. Unal, A. Undrus, G. Unel, F. C. Ungaro, Y. Unno, C. Unverdorben, J. Urban, P. Urquijo, P. Urrejola, G. Usai, L. Vacavant, V. Vacek, B. Vachon, C. Valderanis, E. Valdes Santurio, N. Valencic, S. Valentinetti, A. Valero, L. Valery, S. Valkar, J. A. Valls Ferrer, W. Van Den Wollenberg, P. C. Van Der Deijl, H. van der Graaf, N. van Eldik, P. van Gemmeren, J. Van Nieuwkoop, I. van Vulpen, M. C. van Woerden, M. Vanadia, W. Vandelli, R. Vanguri, A. Vaniachine, P. Vankov, G. Vardanyan, R. Vari, E. W. Varnes, T. Varol, D. Varouchas, A. Vartapetian, K. E. Varvell, J. G. Vasquez, G. A. Vasquez, F. Vazeille, T. Vazquez Schroeder, J. Veatch, V. Veeraraghavan, L. M. Veloce, F. Veloso, S. Veneziano, A. Ventura, M. Venturi, N. Venturi, A. Venturini, V. Vercesi, M. Verducci, W. Verkerke, J. C. Vermeulen, A. Vest, M. C. Vetterli, O. Viazlo, I. Vichou, T. Vickey, O. E. Vickey Boeriu, G. H. A. Viehhauser, S. Viel, L. Vigani, M. Villa, M. Villaplana Perez, E. Vilucchi, M. G. Vincter, V. B. Vinogradov, C. Vittori, I. Vivarelli, S. Vlachos, M. Vlasak, M. Vogel, P. Vokac, G. Volpi, M. Volpi, H. von der Schmitt, E. von Toerne, V. Vorobel, K. Vorobev, M. Vos, R. Voss, J. H. Vossebeld, N. Vranjes, M. Vranjes Milosavljevic, V. Vrba, M. Vreeswijk, R. Vuillermet, I. Vukotic, Z. Vykydal, P. Wagner, W. Wagner, H. Wahlberg, S. Wahrmund, J. Wakabayashi, J. Walder, R. Walker, W. Walkowiak, V. Wallangen, C. Wang, C. Wang, F. Wang, H. Wang, H. Wang, J. Wang, J. Wang, K. Wang, R. Wang, S. M. Wang, T. Wang, T. Wang, W. Wang, X. Wang, C. Wanotayaroj, A. Warburton, C. P. Ward, D. R. Wardrope, A. Washbrook, P. M. Watkins, A. T. Watson, M. F. Watson, G. Watts, S. Watts, B. M. Waugh, S. Webb, M. S. Weber, S. W. Weber, S. A. Weber, J. S. Webster, A. R. Weidberg, B. Weinert, J. Weingarten, C. Weiser, H. Weits, P. S. Wells, T. Wenaus, T. Wengler, S. Wenig, N. Wermes, M. Werner, M. D. Werner, P. Werner, M. Wessels, J. Wetter, K. Whalen, N. L. Whallon, A. M. Wharton, A. White, M. J. White, R. White, D. Whiteson, F. J. Wickens, W. Wiedenmann, M. Wielers, C. Wiglesworth, L. A. M. Wiik-Fuchs, A. Wildauer, F. Wilk, H. G. Wilkens, H. H. Williams, S. Williams, C. Willis, S. Willocq, J. A. Wilson, I. Wingerter-Seez, F. Winklmeier, O. J. Winston, B. T. Winter, M. Wittgen, J. Wittkowski, T. M. H. Wolf, M. W. Wolter, H. Wolters, S. D. Worm, B. K. Wosiek, J. Wotschack, M. J. Woudstra, K. W. Wozniak, M. Wu, M. Wu, S. L. Wu, X. Wu, Y. Wu, T. R. Wyatt, B. M. Wynne, S. Xella, D. Xu, L. Xu, B. Yabsley, S. Yacoob, D. Yamaguchi, Y. Yamaguchi, A. Yamamoto, S. Yamamoto, T. Yamanaka, K. Yamauchi, Y. Yamazaki, Z. Yan, H. Yang, H. Yang, Y. Yang, Z. Yang, W -M. Yao, Y. C. Yap, Y. Yasu, E. Yatsenko, K. H. Yau Wong, J. Ye, S. Ye, I. Yeletskikh, A. L. Yen, E. Yildirim, K. Yorita, R. Yoshida, K. Yoshihara, C. Young, C. J. S. Young, S. Youssef, D. R. Yu, J. Yu, J. M. Yu, J. Yu, L. Yuan, S. P. Y. Yuen, I. Yusuff, B. Zabinski, R. Zaidan, A. M. Zaitsev, N. Zakharchuk, J. Zalieckas, A. Zaman, S. Zambito, L. Zanello, D. Zanzi, C. Zeitnitz, M. Zeman, A. Zemla, J. C. Zeng, Q. Zeng, K. Zengel, O. Zenin, T. Ženiš, D. Zerwas, D. Zhang, F. Zhang, G. Zhang, H. Zhang, J. Zhang, L. Zhang, R. Zhang, R. Zhang, X. Zhang, Z. Zhang, X. Zhao, Y. Zhao, Z. Zhao, A. Zhemchugov, J. Zhong, B. Zhou, C. Zhou, L. Zhou, L. Zhou, M. Zhou, N. Zhou, C. G. Zhu, H. Zhu, J. Zhu, Y. Zhu, X. Zhuang, K. Zhukov, A. Zibell, D. Zieminska, N. I. Zimine, C. Zimmermann, S. Zimmermann, Z. Zinonos, M. Zinser, M. Ziolkowski, L. Živković, G. Zobernig, A. Zoccoli, M. zur Nedden, L. Zwalinski, ATLAS Collaboration

**Affiliations:** 1 Department of Physics, University of Adelaide, Adelaide, Australia; 2 Physics Department, SUNY Albany, Albany, NY USA; 3 Department of Physics, University of Alberta, Edmonton, AB Canada; 4 Department of Physics, Ankara University, Ankara, Turkey; 5 Istanbul Aydin University, Istanbul, Turkey; 6 Division of Physics, TOBB University of Economics and Technology, Ankara, Turkey; 7 LAPP, CNRS/IN2P3 and Université Savoie Mont Blanc, Annecy-le-Vieux, France; 8 High Energy Physics Division, Argonne National Laboratory, Argonne, IL USA; 9 Department of Physics, University of Arizona, Tucson, AZ USA; 10 Department of Physics, The University of Texas at Arlington, Arlington, TX USA; 11 Physics Department, University of Athens, Athens, Greece; 12 Physics Department, National Technical University of Athens, Zografou, Greece; 13 Department of Physics, The University of Texas at Austin, Austin, TX USA; 14 Institute of Physics, Azerbaijan Academy of Sciences, Baku, Azerbaijan; 15 Institut de Física d’Altes Energies (IFAE), The Barcelona Institute of Science and Technology, Barcelona, Spain; 16 Institute of Physics, University of Belgrade, Belgrade, Serbia; 17 Department for Physics and Technology, University of Bergen, Bergen, Norway; 18 Physics Division, Lawrence Berkeley National Laboratory and University of California, Berkeley, CA USA; 19 Department of Physics, Humboldt University, Berlin, Germany; 20 Albert Einstein Center for Fundamental Physics and Laboratory for High Energy Physics, University of Bern, Bern, Switzerland; 21 School of Physics and Astronomy, University of Birmingham, Birmingham, UK; 22 Department of Physics, Bogazici University, Istanbul, Turkey; 23 Department of Physics Engineering, Gaziantep University, Gaziantep, Turkey; 24 Faculty of Engineering and Natural Sciences, Istanbul Bilgi University, Istanbul, Turkey; 25 Faculty of Engineering and Natural Sciences, Bahcesehir University, Istanbul, Turkey; 26 Centro de Investigaciones, Universidad Antonio Narino, Bogotá, Colombia; 27 INFN Sezione di Bologna, Bologna, Italy; 28 Dipartimento di Fisica e Astronomia, Università di Bologna, Bologna, Italy; 29 Physikalisches Institut, University of Bonn, Bonn, Germany; 30 Department of Physics, Boston University, Boston, MA USA; 31 Department of Physics, Brandeis University, Waltham, MA USA; 32 Universidade Federal do Rio De Janeiro COPPE/EE/IF, Rio de Janeiro, Brazil; 33 Electrical Circuits Department, Federal University of Juiz de Fora (UFJF), Juiz de Fora, Brazil; 34 Federal University of Sao Joao del Rei (UFSJ), Sao Joao del Rei, Brazil; 35 Instituto de Fisica, Universidade de Sao Paulo, São Paulo, Brazil; 36 Physics Department, Brookhaven National Laboratory, Upton, NY USA; 37 Transilvania University of Brasov, Brasov, Romania; 38 National Institute of Physics and Nuclear Engineering, Bucharest, Romania; 39 Physics Department, National Institute for Research and Development of Isotopic and Molecular Technologies, Cluj Napoca, Romania; 40 University Politehnica Bucharest, Bucharest, Romania; 41 West University in Timisoara, Timisoara, Romania; 42 Departamento de Física, Universidad de Buenos Aires, Buenos Aires, Argentina; 43 Cavendish Laboratory, University of Cambridge, Cambridge, UK; 44 Department of Physics, Carleton University, Ottawa, ON Canada; 45 CERN, Geneva, Switzerland; 46 Enrico Fermi Institute, University of Chicago, Chicago, IL USA; 47 Departamento de Física, Pontificia Universidad Católica de Chile, Santiago, Chile; 48 Departamento de Física, Universidad Técnica Federico Santa María, Valparaiso, Chile; 49 Institute of High Energy Physics, Chinese Academy of Sciences, Beijing, China; 50 Department of Modern Physics, University of Science and Technology of China, Hefei, Anhui China; 51 Department of Physics, Nanjing University, Nanjing, Jiangsu China; 52 School of Physics, Shandong University, Jinan, Shandong China; 53 Shanghai Key Laboratory for Particle Physics and Cosmology, Department of Physics and Astronomy, Shanghai Jiao Tong University (also affiliated with PKU-CHEP), Shanghai, China; 54 Physics Department, Tsinghua University, Beijing, 100084 China; 55 Laboratoire de Physique Corpusculaire, Clermont Université and Université Blaise Pascal and CNRS/IN2P3, Clermont-Ferrand, France; 56 Nevis Laboratory, Columbia University, Irvington, NY USA; 57 Niels Bohr Institute, University of Copenhagen, Kobenhavn, Denmark; 58 INFN Gruppo Collegato di Cosenza, Laboratori Nazionali di Frascati, Frascati, Italy; 59 Dipartimento di Fisica, Università della Calabria, Rende, Italy; 60 Faculty of Physics and Applied Computer Science, AGH University of Science and Technology, Kraków, Poland; 61 Marian Smoluchowski Institute of Physics, Jagiellonian University, Kraków, Poland; 62 Institute of Nuclear Physics, Polish Academy of Sciences, Kraków, Poland; 63 Physics Department, Southern Methodist University, Dallas, TX USA; 64 Physics Department, University of Texas at Dallas, Richardson, TX USA; 65 DESY, Hamburg and Zeuthen, Germany; 66 Lehrstuh für Experimentelle Physik IV, Technische Universität Dortmund, Dortmund, Germany; 67 Institut für Kern- und Teilchenphysik, Technische Universität Dresden, Dresden, Germany; 68 Department of Physics, Duke University, Durham, NC USA; 69 SUPA-School of Physics and Astronomy, University of Edinburgh, Edinburgh, UK; 70 INFN Laboratori Nazionali di Frascati, Frascati, Italy; 71 Fakultät für Mathematik und Physik, Albert-Ludwigs-Universität, Freiburg, Germany; 72 Section de Physique, Université de Genève, Geneva, Switzerland; 73 INFN Sezione di Genova, Genoa, Italy; 74 Dipartimento di Fisica, Università di Genova, Genoa, Italy; 75 E. Andronikashvili Institute of Physics, Iv. Javakhishvili Tbilisi State University, Tbilisi, Georgia; 76 High Energy Physics Institute, Tbilisi State University, Tbilisi, Georgia; 77 II Physikalisches Institut, Justus-Liebig-Universität Giessen, Giessen, Germany; 78 SUPA-School of Physics and Astronomy, University of Glasgow, Glasgow, UK; 79 II Physikalisches Institut, Georg-August-Universität, Göttingen, Germany; 80 Laboratoire de Physique Subatomique et de Cosmologie, Université Grenoble-Alpes, CNRS/IN2P3, Grenoble, France; 81 Laboratory for Particle Physics and Cosmology, Harvard University, Cambridge, MA USA; 82 Kirchhoff-Institut für Physik, Ruprecht-Karls-Universität Heidelberg, Heidelberg, Germany; 83 Physikalisches Institut, Ruprecht-Karls-Universität Heidelberg, Heidelberg, Germany; 84 ZITI Institut für technische Informatik, Ruprecht-Karls-Universität Heidelberg, Mannheim, Germany; 85 Faculty of Applied Information Science, Hiroshima Institute of Technology, Hiroshima, Japan; 86 Department of Physics, The Chinese University of Hong Kong, Shatin, NT Hong Kong; 87 Department of Physics, The University of Hong Kong, Hong Kong, China; 88 Department of Physics, The Hong Kong University of Science and Technology, Clear Water Bay, Kowloon, Hong Kong, China; 89 Department of Physics, Indiana University, Bloomington, IN USA; 90 Institut für Astro- und Teilchenphysik, Leopold-Franzens-Universität, Innsbruck, Austria; 91 University of Iowa, Iowa City, IA USA; 92 Department of Physics and Astronomy, Iowa State University, Ames, IA USA; 93 Joint Institute for Nuclear Research, JINR Dubna, Dubna, Russia; 94 KEK, High Energy Accelerator Research Organization, Tsukuba, Japan; 95 Graduate School of Science, Kobe University, Kobe, Japan; 96 Faculty of Science, Kyoto University, Kyoto, Japan; 97 Kyoto University of Education, Kyoto, Japan; 98 Department of Physics, Kyushu University, Fukuoka, Japan; 99 Instituto de Física La Plata, Universidad Nacional de La Plata and CONICET, La Plata, Argentina; 100 Physics Department, Lancaster University, Lancaster, UK; 101 INFN Sezione di Lecce, Lecce, Italy; 102 Dipartimento di Matematica e Fisica, Università del Salento, Lecce, Italy; 103 Oliver Lodge Laboratory, University of Liverpool, Liverpool, UK; 104 Department of Physics, Jožef Stefan Institute and University of Ljubljana, Ljubljana, Slovenia; 105 School of Physics and Astronomy, Queen Mary University of London, London, UK; 106 Department of Physics, Royal Holloway University of London, Surrey, UK; 107 Department of Physics and Astronomy, University College London, London, UK; 108 Louisiana Tech University, Ruston, LA USA; 109 Laboratoire de Physique Nucléaire et de Hautes Energies, UPMC and Université Paris-Diderot and CNRS/IN2P3, Paris, France; 110 Fysiska institutionen, Lunds universitet, Lund, Sweden; 111 Departamento de Fisica Teorica C-15, Universidad Autonoma de Madrid, Madrid, Spain; 112 Institut für Physik, Universität Mainz, Mainz, Germany; 113 School of Physics and Astronomy, University of Manchester, Manchester, UK; 114 CPPM, Aix-Marseille Université and CNRS/IN2P3, Marseille, France; 115 Department of Physics, University of Massachusetts, Amherst, MA USA; 116 Department of Physics, McGill University, Montreal, QC Canada; 117 School of Physics, University of Melbourne, Melbourne, VIC Australia; 118 Department of Physics, The University of Michigan, Ann Arbor, MI USA; 119 Department of Physics and Astronomy, Michigan State University, East Lansing, MI USA; 120 INFN Sezione di Milano, Milan, Italy; 121 Dipartimento di Fisica, Università di Milano, Milan, Italy; 122 B.I. Stepanov Institute of Physics, National Academy of Sciences of Belarus, Minsk, Republic of Belarus; 123 National Scientific and Educational Centre for Particle and High Energy Physics, Minsk, Republic of Belarus; 124 Group of Particle Physics, University of Montreal, Montreal, QC Canada; 125 P.N. Lebedev Physical Institute of the Russian, Academy of Sciences, Moscow, Russia; 126 Institute for Theoretical and Experimental Physics (ITEP), Moscow, Russia; 127 National Research Nuclear University MEPhI, Moscow, Russia; 128 D.V. Skobeltsyn Institute of Nuclear Physics, M.V. Lomonosov Moscow State University, Moscow, Russia; 129 Fakultät für Physik, Ludwig-Maximilians-Universität München, Munich, Germany; 130 Max-Planck-Institut für Physik (Werner-Heisenberg-Institut), Munich, Germany; 131 Nagasaki Institute of Applied Science, Nagasaki, Japan; 132 Graduate School of Science and Kobayashi-Maskawa Institute, Nagoya University, Nagoya, Japan; 133 INFN Sezione di Napoli, Naples, Italy; 134 Dipartimento di Fisica, Università di Napoli, Naples, Italy; 135 Department of Physics and Astronomy, University of New Mexico, Albuquerque, NM USA; 136 Institute for Mathematics, Astrophysics and Particle Physics, Radboud University Nijmegen/Nikhef, Nijmegen, The Netherlands; 137 Nikhef National Institute for Subatomic Physics and University of Amsterdam, Amsterdam, The Netherlands; 138 Department of Physics, Northern Illinois University, DeKalb, IL USA; 139 Budker Institute of Nuclear Physics, SB RAS, Novosibirsk, Russia; 140 Department of Physics, New York University, New York, NY USA; 141 Ohio State University, Columbus, OH USA; 142 Faculty of Science, Okayama University, Okayama, Japan; 143 Homer L. Dodge Department of Physics and Astronomy, University of Oklahoma, Norman, OK USA; 144 Department of Physics, Oklahoma State University, Stillwater, OK USA; 145 Palacký University, RCPTM, Olomouc, Czech Republic; 146 Center for High Energy Physics, University of Oregon, Eugene, OR USA; 147 LAL, University of Paris-Sud, CNRS/IN2P3, Université Paris-Saclay, Orsay, France; 148 Graduate School of Science, Osaka University, Osaka, Japan; 149 Department of Physics, University of Oslo, Oslo, Norway; 150 Department of Physics, Oxford University, Oxford, UK; 151 INFN Sezione di Pavia, Pavia, Italy; 152 Dipartimento di Fisica, Università di Pavia, Pavia, Italy; 153 Department of Physics, University of Pennsylvania, Philadelphia, PA USA; 154 National Research Centre “Kurchatov Institute” B.P. Konstantinov Petersburg Nuclear Physics Institute, St. Petersburg, Russia; 155 INFN Sezione di Pisa, Pisa, Italy; 156 Dipartimento di Fisica E. Fermi, Università di Pisa, Pisa, Italy; 157 Department of Physics and Astronomy, University of Pittsburgh, Pittsburgh, PA USA; 158 Laboratório de Instrumentação e Física Experimental de Partículas-LIP, Lisbon, Portugal; 159 Faculdade de Ciências, Universidade de Lisboa, Lisbon, Portugal; 160 Department of Physics, University of Coimbra, Coimbra, Portugal; 161 Centro de Física Nuclear da Universidade de Lisboa, Lisbon, Portugal; 162 Departamento de Fisica, Universidade do Minho, Braga, Portugal; 163 Departamento de Fisica Teorica y del Cosmos and CAFPE, Universidad de Granada, Granada, Spain; 164 Dep Fisica and CEFITEC of Faculdade de Ciencias e Tecnologia, Universidade Nova de Lisboa, Caparica, Portugal; 165 Institute of Physics, Academy of Sciences of the Czech Republic, Prague, Czech Republic; 166 Czech Technical University in Prague, Prague, Czech Republic; 167 Faculty of Mathematics and Physics, Charles University in Prague, Prague, Czech Republic; 168 State Research Center Institute for High Energy Physics (Protvino), NRC KI, Protvino, Russia; 169 Particle Physics Department, Rutherford Appleton Laboratory, Didcot, UK; 170 INFN Sezione di Roma, Rome, Italy; 171 Dipartimento di Fisica, Sapienza Università di Roma, Rome, Italy; 172 INFN Sezione di Roma Tor Vergata, Rome, Italy; 173 Dipartimento di Fisica, Università di Roma Tor Vergata, Rome, Italy; 174 INFN Sezione di Roma Tre, Rome, Italy; 175 Dipartimento di Matematica e Fisica, Università Roma Tre, Rome, Italy; 176 Faculté des Sciences Ain Chock, Réseau Universitaire de Physique des Hautes Energies-Université Hassan II, Casablanca, Morocco; 177 Centre National de l’Energie des Sciences Techniques Nucleaires, Rabat, Morocco; 178 Faculté des Sciences Semlalia, Université Cadi Ayyad, LPHEA-Marrakech, Marrakech, Morocco; 179 Faculté des Sciences, Université Mohamed Premier and LPTPM, Oujda, Morocco; 180 Faculté des Sciences, Université Mohammed V, Rabat, Morocco; 181 DSM/IRFU (Institut de Recherches sur les Lois Fondamentales de l’Univers), CEA Saclay (Commissariat à l’Energie Atomique et aux Energies Alternatives), Gif-sur-Yvette, France; 182 Santa Cruz Institute for Particle Physics, University of California Santa Cruz, Santa Cruz, CA USA; 183 Department of Physics, University of Washington, Seattle, WA USA; 184 Department of Physics and Astronomy, University of Sheffield, Sheffield, UK; 185 Department of Physics, Shinshu University, Nagano, Japan; 186 Fachbereich Physik, Universität Siegen, Siegen, Germany; 187 Department of Physics, Simon Fraser University, Burnaby, BC Canada; 188 SLAC National Accelerator Laboratory, Stanford, CA USA; 189 Faculty of Mathematics, Physics and Informatics, Comenius University, Bratislava, Slovak Republic; 190 Department of Subnuclear Physics, Institute of Experimental Physics of the Slovak Academy of Sciences, Kosice, Slovak Republic; 191 Department of Physics, University of Cape Town, Cape Town, South Africa; 192 Department of Physics, University of Johannesburg, Johannesburg, South Africa; 193 School of Physics, University of the Witwatersrand, Johannesburg, South Africa; 194 Department of Physics, Stockholm University, Stockholm, Sweden; 195 The Oskar Klein Centre, Stockholm, Sweden; 196 Physics Department, Royal Institute of Technology, Stockholm, Sweden; 197 Departments of Physics and Astronomy and Chemistry, Stony Brook University, Stony Brook, NY USA; 198 Department of Physics and Astronomy, University of Sussex, Brighton, UK; 199 School of Physics, University of Sydney, Sydney, Australia; 200 Institute of Physics, Academia Sinica, Taipei, Taiwan; 201 Department of Physics, Technion: Israel Institute of Technology, Haifa, Israel; 202 Raymond and Beverly Sackler School of Physics and Astronomy, Tel Aviv University, Tel Aviv, Israel; 203 Department of Physics, Aristotle University of Thessaloniki, Thessaloniki, Greece; 204 International Center for Elementary Particle Physics and Department of Physics, The University of Tokyo, Tokyo, Japan; 205 Graduate School of Science and Technology, Tokyo Metropolitan University, Tokyo, Japan; 206 Department of Physics, Tokyo Institute of Technology, Tokyo, Japan; 207 Department of Physics, University of Toronto, Toronto, ON Canada; 208 TRIUMF, Vancouver, BC Canada; 209 Department of Physics and Astronomy, York University, Toronto, ON Canada; 210 Faculty of Pure and Applied Sciences, and Center for Integrated Research in Fundamental Science and Engineering, University of Tsukuba, Tsukuba, Japan; 211 Department of Physics and Astronomy, Tufts University, Medford, MA USA; 212 Department of Physics and Astronomy, University of California Irvine, Irvine, CA USA; 213 INFN Gruppo Collegato di Udine, Sezione di Trieste, Udine, Italy; 214 ICTP, Trieste, Italy; 215 Dipartimento di Chimica Fisica e Ambiente, Università di Udine, Udine, Italy; 216 Department of Physics and Astronomy, University of Uppsala, Uppsala, Sweden; 217 Department of Physics, University of Illinois, Urbana, IL USA; 218 Instituto de Fisica Corpuscular (IFIC) and Departamento de Fisica Atomica, Molecular y Nuclear and Departamento de Ingeniería Electrónica and Instituto de Microelectrónica de Barcelona (IMB-CNM), University of Valencia and CSIC, Valencia, Spain; 219 Department of Physics, University of British Columbia, Vancouver, BC Canada; 220 Department of Physics and Astronomy, University of Victoria, Victoria, BC Canada; 221 Department of Physics, University of Warwick, Coventry, UK; 222 Waseda University, Tokyo, Japan; 223 Department of Particle Physics, The Weizmann Institute of Science, Rehovot, Israel; 224 Department of Physics, University of Wisconsin, Madison, WI USA; 225 Fakultät für Physik und Astronomie, Julius-Maximilians-Universität, Würzburg, Germany; 226 Fakultät für Mathematik und Naturwissenschaften, Fachgruppe Physik, Bergische Universität Wuppertal, Wuppertal, Germany; 227 Department of Physics, Yale University, New Haven, CT USA; 228 Yerevan Physics Institute, Yerevan, Armenia; 229 Centre de Calcul de l’Institut National de Physique Nucléaire et de Physique des Particules (IN2P3), Villeurbanne, France; 230 CERN, 1211 Geneva 23, Switzerland

## Abstract

A search for neutral Higgs bosons of the minimal supersymmetric standard model (MSSM) and for a heavneutral $Z^{\prime }$ boson is performed using a data sample corresponding to an integrated luminosity of 3.2 fb$^{-1}$ from proton–proton collisions at $\sqrt{s} = 13$  ${\mathrm {TeV}}$ recorded by the ATLAS detector at the LHC. The heavy resonance is assumed to decay to a $\tau ^+ \tau ^-$ pair with at least one $\tau$ lepton decaying to final states with hadrons and a neutrino. The search is performed in the mass range of 0.2–1.2  ${\mathrm {TeV}}$ for the MSSM neutral Higgs bosons and 0.5–2.5  ${\mathrm {TeV}}$ for the heavy neutral $Z^{\prime }$ boson. The data are in good agreement with the background predicted by the Standard Model. The results are interpreted in MSSM and $Z^{\prime }$ benchmark scenarios. The most stringent constraints on the MSSM $m_A$–$\tan \beta$ space exclude at 95 % confidence level (CL) $\tan \beta > 7.6$ for $m_A = 200$ $\text {GeV}$ in the $m_{h}^{\text {mod+}}$ MSSM scenario. For the Sequential Standard Model, a $Z^{\prime }_\mathrm {SSM}$ mass up to 1.90  ${\mathrm {TeV}}$ is excluded at 95 % CL and masses up to 1.82–2.17  ${\mathrm {TeV}}$ are excluded for a $Z^{\prime }_{\mathrm {SFM}}$ of the strong flavour model.

## Introduction

The discovery of a scalar particle at the LHC [1, 2] has provided important insight into the mechanism of electroweak symmetry breaking. Experimental studies of the new particle [3–7] demonstrate consistency with the standard model (SM) Higgs boson [8–13]. However, it remains possible that the discovered particle is part of an extended scalar sector, a scenario that is favoured by a number of theoretical arguments [14, 15].

The minimal supersymmetric standard model (MSSM) [16–20] is the simplest extension of the SM that includes supersymmetry. The MSSM requires two Higgs doublets of opposite hypercharge. Assuming that CP symmetry is conserved, this results in one CP-odd (*A*) and two CP-even (*h*, *H*) neutral Higgs bosons and two charged Higgs bosons ($H^{\pm }$). At tree level, the properties of the Higgs sector in the MSSM depend on only two non-SM parameters, which can be chosen to be the mass of the CP-odd Higgs boson, $m_A$, and the ratio of the vacuum expectation values of the two doublets, $\tan \beta$. Beyond tree level, additional parameters affect the Higgs sector, the choice of which defines various MSSM benchmark scenarios. In some scenarios, such as $m_{h}^{\text {mod}+}$ [21], the top-squark mixing parameter is chosen such that the mass of the lightest CP-even Higgs boson, $m_h$, is close to the measured mass of the Higgs boson that was discovered at the LHC. A different approach is employed in the hMSSM scenario [22, 23] in which the value of $m_h$ can be used, with certain assumptions, to predict the remaining masses and couplings of the MSSM Higgs bosons without explicit reference to the soft supersymmetry-breaking parameters. The couplings of the MSSM heavy Higgs bosons to down-type fermions are enhanced with respect to the SM for large $\tan \beta$ values, resulting in increased branching fractions to $\tau$ leptons and *b*-quarks,^1^ as well as a higher cross section for Higgs boson production in association with *b*-quarks. This has motivated a variety of searches for a scalar boson in $\tau \tau$ and *bb* final states at LEP [24], the Tevatron [25–27] and the LHC [28–32].

Heavy $Z^\prime$ gauge bosons appear in several models [33–37] and are a common extension of the SM [38]. Such $Z^\prime$ bosons can appear in theories extending the electroweak gauge group, where lepton universality is typically conserved. A frequently used benchmark is the sequential standard model (SSM) [39], which contains a single additional $Z^\prime$ boson with the same couplings as the SM *Z* boson. Some models offering an explanation for the high mass of the top-quark, predict instead that such bosons couple preferentially to third-generation fermions [40–43]. A model predicting additional weak gauge bosons $Z^\prime$ and $W^\prime$ coupling preferentially to third-generation fermions is the strong flavour model (SFM) [41, 43].

**Fig. 1:**
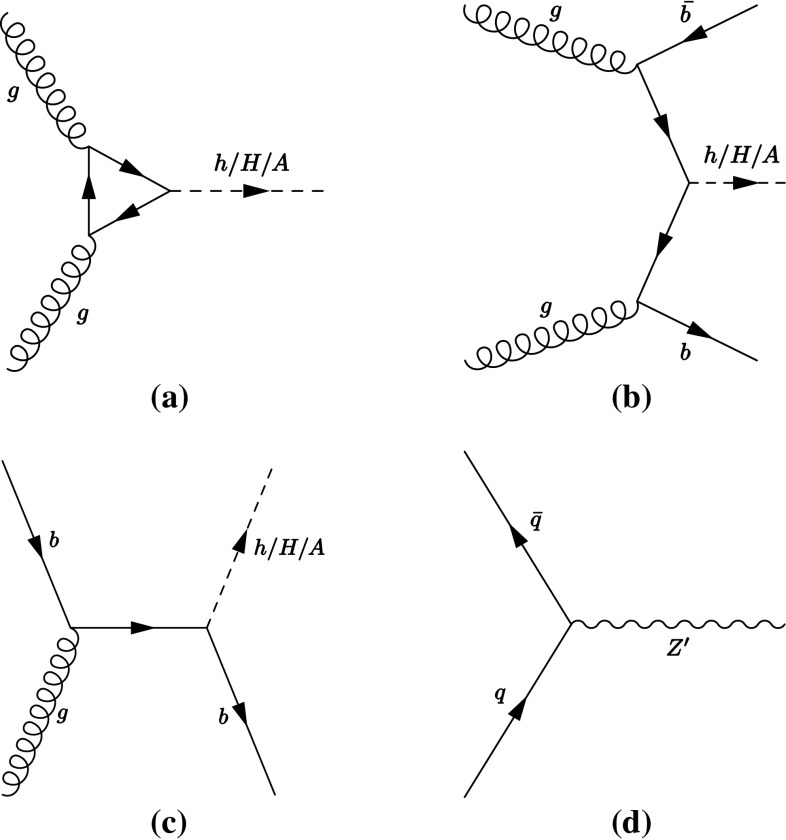
Lowest-order Feynman diagrams for **a** gluon–gluon fusion and *b*-associated production in the **b** four-flavour and **c** five-flavour schemes of a neutral MSSM Higgs boson. Feynman diagram for Drell–Yan production of a $Z^\prime$ boson at lowest order (**d**)

Direct searches for high-mass resonances decaying to $\tau \tau$ have been performed by the ATLAS [44] and CMS [45] collaborations using 5 $\mathrm{fb}^{-1}$ of integrated luminosity at $\sqrt{s}=7~~{\mathrm {TeV}}$. ATLAS [46] updated the search with 20 $\mathrm{fb}^{-1}$ of integrated luminosity at $\sqrt{s}=8~~{\mathrm {TeV}}$. Indirect limits on $Z^\prime$ bosons with non-universal flavour couplings have been set based on measurements from LEP [47].

This paper presents the results of a search for neutral MSSM Higgs bosons as well as high-mass $Z^\prime$ resonances in the $\tau \tau$ decay mode using 3.2 fb$^{-1}$ of proton–proton collision data collected with the ATLAS detector [48] in 2015 at a centre-of-mass energy of 13  ${\mathrm {TeV}}$. The search is performed for the $\tau _{\mathrm {lep}}\tau _{\mathrm {had}}$ and $\tau _{\mathrm {had}}\tau _{\mathrm {had}}$ decay modes, where $\tau _{\mathrm {lep}}$ represents the decay of a $\tau$ lepton to an electron or a muon and neutrinos and $\tau _{\mathrm {had}}$ represents the decay to one or more hadrons and a neutrino. The search considers narrow resonances in the mass range of 0.2–1.2  ${\mathrm {TeV}}$ and $\tan \beta$ range of 1–60 for the MSSM Higgs bosons. For the $Z^\prime$ boson search, the mass range of 0.5–2.5  ${\mathrm {TeV}}$ is considered. Higgs boson production through gluon–gluon fusion and in association with *b*-quarks is considered (Fig. 1a–1c), with the latter mode dominating for high $\tan \beta$ values. Hence both the $\tau _{\mathrm {lep}}\tau _{\mathrm {had}}$ and $\tau _{\mathrm {had}}\tau _{\mathrm {had}}$ channels are split into *b*-tag and *b*-veto categories, based on the presence or absence of jets originating from *b*-quarks in the final state. Since a $Z^\prime$ boson is expected to be predominantly produced via a Drell–Yan process (Fig. 1d), there is little gain in splitting the data into *b*-tag and *b*-veto categories. Hence, the $Z^\prime$ analysis uses an inclusive selection instead.

## Data sample and Monte Carlo simulation

The ATLAS detector [48] at the LHC consists of an inner tracking detector with a coverage in pseudorapidity^2^ up to $|\eta |~=~2.5$ surrounded by a thin superconducting solenoid providing a 2 T axial magnetic field, electromagnetic and hadronic calorimeters extending up to $|\eta |~=~4.9$ and a muon spectrometer covering $|\eta |~<~2.7$. A new innermost layer was added to the pixel tracking detector after the end Run-1 at a radial distance of 3.3 cm from the beam line [49, 50]. The ATLAS trigger system consists of a hardware-based first level trigger, followed by a software-based high-level trigger (HLT). The integrated luminosity used in this search, considering the data-taking periods of 2015 in which all relevant detector subsystems were operational, is 3.2 fb$^{-1}$. The luminosity measurement and its uncertainty are derived following a methodology similar to that detailed in Ref. [51], from a calibration of the luminosity scale using *x*–*y* beam-separation scans performed in August 2015.

Simulated events with a heavy neutral MSSM Higgs boson produced via gluon–gluon fusion and in association with *b*-quarks are generated with the POWHEG-BOX v2 [52–54] and MADGRAPH5_aMC@NLO 2.1.2 [55, 56] programs, respectively. The CT10 [57] and CT10nlo_nf4 [58] sets of parton distribution functions (PDFs) are used, respectively. PYTHIA 8.210 [59] with the AZNLO [60] (A14 [61]) set of tuned parameters, or “tune”, is used for the parton shower, underlying event and hadronization in the gluon–gluon fusion (*b*-associated) production. The production cross sections for the various MSSM scenarios are calculated using SusHi [62] for gluon fusion production [63–75] and *b*-associated production in the five-flavour scheme [76]; *b*-associated production in the four-flavour scheme is calculated according to Refs. [77, 78]. The final *b*-associated production cross section is obtained by using the method in Ref. [79] to match the four-flavour and five-flavour scheme cross sections. The masses and the couplings of the Higgs bosons are computed with FeynHiggs [80–84], whereas the branching fraction calculation follows the procedure described in Ref. [85]. In the case of the hMSSM scenario, the procedure described in Ref. [23] is followed for the production cross sections and HDECAY [86] is used for the branching fraction calculation.

The $Z^\prime {}$ signals are simulated by reweighting a leading-order (LO) $Z/\gamma ^{*}\rightarrow \tau \tau$ sample using the TauSpinner algorithm [87–89] to account for spin effects in the $\tau$ decays. The $Z/\gamma ^{*}\rightarrow \tau \tau$ sample, enriched with high invariant mass events, is generated with PYTHIA 8.165 [90] using the NNPDF2.3LO PDF set [91] and the A14 tune for the underlying event. Interference between the $Z^\prime$ signals and the SM $Z/\gamma ^{*}$ production is not included.

The simulated backgrounds consist of the production of *Z*+jets, *W*+jets, $t\bar{t}$ pairs, single top quarks and electroweak dibosons (*WW* / *WZ* / *ZZ*). These are modelled with several event generators as described below, while contributions from multi-jet production are estimated with data as described in Sect. 5.

Simulated samples of *Z*+jets events for the $\tau _{\mathrm {lep}}\tau _{\mathrm {had}}$ and $\tau _{\mathrm {had}}\tau _{\mathrm {had}}$ channels and *W*+jets events for the $\tau _{\mathrm {lep}}\tau _{\mathrm {had}}$ channel are produced using POWHEG-BOX v2 interfaced to PYTHIA 8.186 with the AZNLO tune. In this sample, PHOTOS++ v3.52 [92, 93] is used for final-state QED radiation. A dedicated *W*+jets sample binned in $p_{\text {T}} ^{W}$, produced using the SHERPA 2.1.1 generator [94], is used in the $\tau _{\mathrm {had}}\tau _{\mathrm {had}}$ channel in order to enhance the number of events with high invariant mass. For this sample, matrix elements are calculated for up to two partons at next-to-leading order (NLO) and four partons at LO, merged with the SHERPA parton shower model using the ME+PS@NLO prescription [95]. Spin correlation effects between the *W* boson and its decay products are simulated with the TauSpinner program. All *W*/*Z*+jets samples use the CT10 PDF set and are normalized to the next-to-next-to-leading-order (NNLO) cross sections calculated using FEWZ [96–98].

The POWHEG-BOX v2 program with the CT10 PDF set is used for the generation of $t\bar{t}$ pairs and single top quarks in the *Wt*- and *s*-channels. Samples of *t*-channel single-top-quark events are produced with the POWHEG-BOX v1 generator employing the four-flavour scheme for the NLO matrix element calculations together with the fixed four-flavour scheme PDF set CT10f4; the top-quark decay is simulated with MadSpin [99]. For all samples of top-quark production, the spin correlations are preserved and the parton shower, fragmentation and underlying event are simulated using PYTHIA 6.428 [100] with the CTQ6L1 PDF set and the corresponding Perugia 2012 tune [101]. Final-state QED radiation is simulated using PHOTOS++ v3.52. The top-quark mass is set to 172.5 $\text {GeV}$. The $t\bar{t}$ production sample is normalized to the NNLO cross section, including soft-gluon resummation to next-to-next-to-leading-logarithm accuracy (Ref. [102] and references therein). The normalization of the single-top-quark event samples uses an approximate NNLO calculation from Refs. [103–105].

Finally, diboson processes are simulated using the SHERPA 2.1.1 program with the CT10 PDF. They are calculated for up to one additional parton at NLO, depending on the process, and up to three additional partons at LO. The diboson samples use the NLO cross sections SHERPA calculates.

The simulation of *b*- and *c*-hadron decays for all samples, excluding those generated with SHERPA, uses EvtGen v1.2.0 [106]. All simulated samples include the effect of multiple proton-proton interactions in the same and neighbouring bunch crossings (“pile-up”) by overlaying simulated minimum-bias events on each generated signal or background event. These minimum-bias events are generated with PYTHIA 8.186 [90, 100], using the A2 tune [107] and the MSTW2008LO PDF [108]. Each sample is simulated using the full GEANT4 [109, 110] simulation of the ATLAS detector, with the exception of the *b*-associated MSSM Higgs boson signal, for which the ATLFAST-II [110, 111] fast simulation framework is used. Finally, the Monte Carlo (MC) samples are processed through the same reconstruction software as for the data.

## Object reconstruction and identification

The primary vertex of each event is chosen as the proton?proton vertex candidate with the highest sum of the squared transverse momenta of all associated tracks. Electron candidates are reconstructed from energy deposits in the electromagnetic calorimeter associated with a charged-particle track measured in the inner detector. The final electron candidates are required to pass the “loose” likelihood-based identification selection [112, 113], to have a transverse energy $E_{\text {T}} > 15$ $\text {GeV}$ and to be in the fiducial volume of the inner detector, $|\eta |<2.47$. The transition region between the barrel and end-cap calorimeters ($1.37<|\eta |<1.52$) is excluded.

Muon candidates are reconstructed from track segments in the muon spectrometer, matched with tracks found in the inner detector within $|\eta |<2.5$. The tracks of the final muon candidates are refit using the complete track information from both detector systems and are required to have a transverse momentum $p_{\text {T}} >15$ $\text {GeV}$ and to pass the “loose” muon identification requirements [114].

Both the electrons and muons are required to pass a $p_{\text {T}}$-dependent isolation selection, which utilizes both calorimetric and tracking information, with an efficiency of 90 % (99 %) for transverse momentum of $p_{\text {T}} =25~(60)$ $\text {GeV}$. The isolation provides an efficiency that grows as a function of lepton $p_{\text {T}}$, since the background from jets faking leptons becomes less important as the lepton $p_{\text {T}}$ increases. The contributions from pile-up and the underlying event activity are corrected on an event-by-event basis using the ambient energy density technique [115].

Jets are reconstructed from topological clusters [116] in the calorimeter using the anti-$k_t$ algorithm [117], with a radius parameter value $R =0.4$. To reduce the effect of pile-up, a jet vertex tagger algorithm is used for jets with $p_{\text {T}} < 50$ $\text {GeV}$ and $|\eta | < 2.4$. It employs a multivariate technique based on jet energy, vertexing and tracking variables to determine the likelihood that the jet originates from pile-up [118]. In order to identify jets containing *b*-hadrons (*b*-jets), a multivariate algorithm is used  [119, 120]. A working point that corresponds to an average efficiency of 70 % for *b*-jets in $t\bar{t}$ simulated events is chosen. The misidentification rates for *c*-jets, $\tau$-jets and jets initiated by light quarks or gluons for the same working point and in the same sample of simulated $t\bar{t}$ events are approximately 10, 4 and 0.2 % respectively.

Hadronic decays of $\tau$ leptons are predominantly characterized by the presence of one or three charged particles, accompanied by a neutrino and possibly neutral pions. The reconstruction of the visible decay products, hereafter referred to as $\tau _{\mathrm {had-vis}}$, starts with jets with $p_{\text {T}} >10$ $\text {GeV}$. The $\tau _{\mathrm {had-vis}}$ candidate must have energy deposits in the calorimeters in the range $|\eta | < 2.5$, with the transition region between the barrel and end-cap calorimeters excluded. Additionally, they must have $p_{\text {T}} > 20~\text {GeV}$, one or three associated tracks and an electric charge of $\pm 1$. A multivariate boosted decision tree (BDT) identification, based on calorimetric shower shape and track multiplicity of the $\tau _{\mathrm {had-vis}}$ candidates, is used to reject backgrounds from jets. In this analysis, two $\tau _{\mathrm {had-vis}}$ identification criteria are used: “loose” and “medium” with efficiencies measured in $Z\rightarrow \tau \tau$ decays of about 60 % (50 %) and 55 % (40 %) for one-track (three-track) $\tau _{\mathrm {had-vis}}$ candidates, respectively [121]. An additional dedicated likelihood-based veto is used to reduce the number of electrons misidentified as $\tau _{\mathrm {had-vis}}$.

Signals in the detector can be used in more than one reconstructed object. Objects that have a geometric overlap are removed according to the following priorities:

- Jets within a $\Delta R = 0.2$ cone around a selected $\tau _{\mathrm {had-vis}}$ are excluded.
- Jets within a $\Delta R = 0.4$ cone around an electron or muon are excluded.
- Any $\tau _{\mathrm {had-vis}}$ within a $\Delta R = 0.2$ cone around an electron or muon is excluded.
- Electrons within a $\Delta R$ = 0.2 cone around a muon are excluded.

The missing transverse momentum ($E_{\text {T}}^{\text {miss}}$) is calculated as the modulus of the negative vectorial sum of the $\mathbf {p_{\text {T}}}$ of all fully reconstructed and calibrated jets and leptons [122]. This procedure includes a “soft term”, which is calculated based on the inner-detector tracks originating from the primary vertex that are not associated to reconstructed objects.

## Search channels

### $\tau _{\mathrm {lep}}\tau _{\mathrm {had}}$ channel

Events in the $\tau _{\mathrm {lep}}\tau _{\mathrm {had}}$ channel are recorded using single-muon triggers and a logical-OR combination of single-electron triggers. Single-electron triggers with $p_{\text {T}}$ thresholds of $24~\text {GeV}$, $60~\text {GeV}$ and $120~\text {GeV}$ are used for the $\tau _e\tau _{\mathrm {had}}$ channel. For the $\tau _\mu \tau _{\mathrm {had}}$ channel, a single-muon trigger with a $p_{\text {T}}$ threshold of $50~\text {GeV}$ is used if the muon $p_{\text {T}}$ is larger than $55~\text {GeV}$ and a single-muon trigger with a $p_{\text {T}}$ threshold of $20~\text {GeV}$ is used otherwise. The triggers impose electron and muon quality requirements which are tighter for the triggers with lower $p_{\text {T}}$ thresholds.

Events must have at least one identified $\tau _{\mathrm {had-vis}}$ candidate and either one electron or one muon candidate which is geometrically matched to the HLT object that triggered the event. Events with more than one electron or muon fulfilling the criteria described in Sect. 3 are rejected in order to reduce the backgrounds from $Z/\gamma ^{*}\rightarrow \ell \ell$ production, where $\ell = e$, $\mu$. The selected lepton must have a transverse momentum $p_{\text {T}} >30\,\text {GeV}$ and pass the “medium” identification requirement.

The $\tau _{\mathrm {had-vis}}$ candidate is required to have $p_{\text {T}} > 25~\text {GeV}$, pass the “medium” BDT-based identification requirement and lie in the range $|\eta |<2.3$. The latter requirement is motivated by a larger rate of electrons misidentified as $\tau _{\mathrm {had-vis}}$ candidates at higher $|\eta |$ values: the rate is above 10 % for $|\eta |>2.3$, while it ranges from 0.5 to 3 % for lower $|\eta |$ values. If there is more than one $\tau _{\mathrm {had-vis}}$ candidate, the candidate with the highest $p_{\text {T}}$ is selected and the others are treated as jets. Finally, the identified lepton and the $\tau _{\mathrm {had-vis}}$ are required to have opposite electric charge.

Subsequently, the following selection requirements are applied:

- $$\Delta \phi (\tau _{\mathrm {had-vis}}, \ell ) > 2.4$$.
- $$m_\mathrm{T}(\ell ,E_{\text {T}}^{\text {miss}}) \equiv \sqrt{2p_{\text {T}} (\ell )E_{\text {T}}^{\text {miss}} \big [1-\cos \Delta \phi (\ell ,E_{\text {T}}^{\text {miss}})\big ]} < 40~\text {GeV}$$.
- For the $\tau _{e}\tau _{\mathrm {had}}$ channel, events are vetoed if the invariant mass of the electron and the visible $\tau$ lepton decay products is in the range $80<m_{\text {vis}}(e,\tau _{\mathrm {had-vis}})<110~\text {GeV}$.

**Fig. 2:**
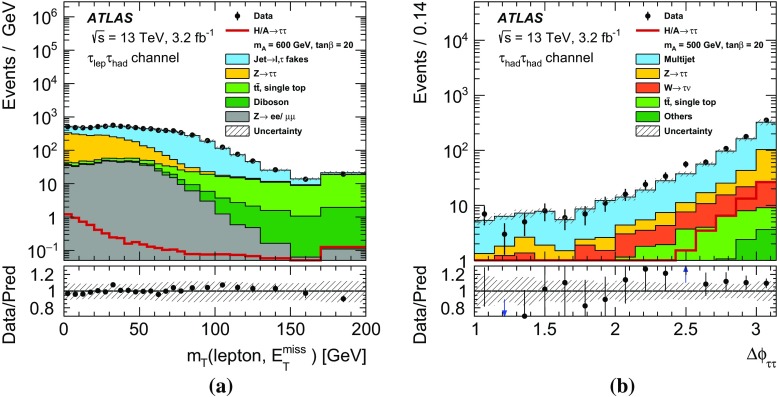
The distributions of **a** $m_\mathrm{T}(\ell ,E_{\text {T}}^{\text {miss}})$ in the $\tau _{\mathrm {lep}}\tau _{\mathrm {had}}$ channel and **b** $\Delta \phi (\tau _{\mathrm {had-vis},1}, \tau _{\mathrm {had-vis},2})$ for the $\tau _{\mathrm {had}}\tau _{\mathrm {had}}$ channel for the inclusive selection with the criterion for the variable displayed removed. The label “Others” in **b** refers to contributions due to diboson, $Z(\rightarrow \ell \ell )$+jets and $W(\rightarrow \ell \nu )$+jets production. Bins have a varying size and overflows are included in the last bin of the distribution on the *left*

The requirement on $\Delta \phi (\tau _{\mathrm {had-vis}}, \ell )$ gives an overall reduction of SM backgrounds with little signal loss. The requirement on $m_\mathrm{T}(\ell ,E_{\text {T}}^{\text {miss}})$, the distribution of which is shown in Fig. 2a, serves to remove events that originate from processes containing a *W* boson: in signal events, the missing transverse momentum is usually in the same direction as the $\tau _{\mathrm {lep}}$, resulting in a low value of $m_\mathrm{T}(\ell ,E_{\text {T}}^{\text {miss}})$. The requirement on $m_{\text {vis}}(e,\tau _{\mathrm {had-vis}})$ reduces the contribution of $Z\rightarrow ee$ decays, where an electron is misidentified as a $\tau _{\mathrm {had-vis}}$ candidate. These selection criteria define the inclusive $\tau _{\mathrm {lep}}\tau _{\mathrm {had}}$ selection.

### $\tau _{\mathrm {had}}\tau _{\mathrm {had}}$ channel

Events in the $\tau _{\mathrm {had}}\tau _{\mathrm {had}}$ channel are selected by a trigger that requires a single $\tau _{\mathrm {had-vis}}$ satisfying the “medium” $\tau _{\mathrm {had-vis}}$ identification criterion with $p_{\text {T}} >80$ $\text {GeV}$. The leading $\tau _{\mathrm {had-vis}}$ candidate in $p_{\text {T}}$ must geometrically match the HLT object. A $p_{\text {T}}$ requirement is applied to the leading $\tau _{\mathrm {had-vis}}$ candidate, $p_{\text {T}} >110$ $\text {GeV}$, and to the sub-leading $\tau _{\mathrm {had-vis}}$ candidate, $p_{\text {T}} >55$ $\text {GeV}$. Furthermore, the leading (sub-leading) $\tau _{\mathrm {had-vis}}$ candidate has to satisfy the “medium” (“loose”) $\tau _{\mathrm {had-vis}}$ identification criterion. Events with electrons or muons fulfilling the loose selection criteria described in Sect. 3 (with the exception of the isolation requirement) are vetoed to reduce electroweak background processes and guarantee orthogonality with the $\tau _{\mathrm {lep}}\tau _{\mathrm {had}}$ channel.

The leading and sub-leading $\tau _{\mathrm {had-vis}}$ candidates must have opposite electric charge and have a back-to-back topology in the transverse plane, $\Delta \phi (\tau _{\mathrm {had-vis},1}, \tau _{\mathrm {had-vis},2})>2.7$. The distribution of $\Delta \phi (\tau _{\mathrm {had-vis},1}, \tau _{\mathrm {had-vis},2})$ before this requirement is shown in Fig. 2b. This selection defines the inclusive $\tau _{\mathrm {had}}\tau _{\mathrm {had}}$ selection.

### Event categories

In the search for $Z^\prime$ bosons, the event selections described in Sects. 4.1 and 4.2 result in a signal selection efficiency^3^ varying between 0.8 % (2.0 %) at $m_{Z^\prime }=500~ \text {GeV}$ and 3.4 % (3.8 %) at $m_{Z^\prime }=2.5 ~{\mathrm {TeV}}$ for the $\tau _{\mathrm {lep}}\tau _{\mathrm {had}}$ ($\tau _{\mathrm {had}}\tau _{\mathrm {had}}$) channel. In the search for the *H* / *A* bosons, events satisfying the inclusive selection are categorized to exploit the two different signal production modes as follows:

- *b*-veto: no *b*-tag jets in the event,
- *b*-tag: at least one *b*-tag jet in the event.

In the $b$-veto category, the *H* / *A* signal selection efficiency varies between 2 % at $m_A=200\,\text {GeV}$ and 7 % at $m_A=1.2\,~{\mathrm {TeV}}$ for the gluon–gluon fusion production mode in the $\tau _{\mathrm {lep}}\tau _{\mathrm {had}}$ channel, and from 0.1 to 15 % in the $\tau _{\mathrm {had}}\tau _{\mathrm {had}}$ channel for the same mass range. In the $b\text {-tagging}$ category, the signal selection efficiency varies from 0.5 % at $m_A=200~\text {GeV}$ to  2% at $m_A=1.2~~{\mathrm {TeV}}$ for the *b*-associated production mode in the $\tau _{\mathrm {lep}}\tau _{\mathrm {had}}$ channel, and from 0.1 to 6 % in the $\tau _{\mathrm {had}}\tau _{\mathrm {had}}$ channel.

### Di-tau mass reconstruction

The di-tau mass reconstruction is critical in achieving good separation between signal and background. However, its reconstruction is challenging due to the presence of neutrinos from the $\tau$ lepton decays. The mass reconstruction used for both the $\tau _{\mathrm {had}}\tau _{\mathrm {had}}$ and $\tau _{\mathrm {lep}}\tau _{\mathrm {had}}$ channels is the total transverse mass, defined as

1 $$\begin{aligned} m_\mathrm{T}^{\text {tot}} = \sqrt{ m_\mathrm{T}^{2}(E_{\text {T}}^{\text {miss}}, \tau _1) + m_\mathrm{T}^{2}(E_{\text {T}}^{\text {miss}}, \tau _2) + m_\mathrm{T}^{2}(\tau _1, \tau _2)}, \end{aligned}$$

where $m_\mathrm{T}(a, b)$ is defined as

2 $$\begin{aligned} m_\mathrm{T}(a, b) = \sqrt{2p_{\text {T}} (a) p_{\text {T}} (b) [1-\cos \Delta \phi (a, b)]} \end{aligned}$$

and $\tau$ refers to the visible decay of the $\tau$ lepton ($\ell$ or $\tau _{\mathrm {had-vis}}$). More complex mass reconstruction techniques were investigated, but they did not improve the expected sensitivity.

## Background estimation

The background processes can be categorized according to whether the electron/muon and/or the $\tau _{\mathrm {had-vis}}$ are correctly identified. Backgrounds from processes with correctly identified $\tau _{\mathrm {had-vis}}$, electrons and muons, or where the $\tau _{\mathrm {had-vis}}$ is due to a misidentified electron/muon in the $\tau _{\mathrm {lep}}\tau _{\mathrm {had}}$ channel, are estimated from simulation. Data-driven techniques are used for processes where the $\tau _{\mathrm {had-vis}}$, or both the lepton and $\tau _{\mathrm {had-vis}}$ are misidentified. The background contributions originating from processes where only the lepton is misidentified are found to be negligible.

**Table 1:** Description of the control regions used in the $\tau _{\mathrm {lep}}\tau _{\mathrm {had}}$ and $\tau _{\mathrm {had}}\tau _{\mathrm {had}}$ channels

$\tau _{\mathrm {lep}}\tau _{\mathrm {had}}$ signal region	$\Delta \phi (\tau , \ell ) > 2.4$, $m_\mathrm{T}(\ell ,E_{\text {T}}^{\text {miss}}) < 40~\text {GeV}$,
	Veto $80< m_{e,\tau } < 110~\text {GeV}$ for $\tau _e\tau _{\mathrm {had}}$,
	$N_{b\text {-tag}}\ge 1$ (*b*-tag category), $N_{b\text {-tag}}= 0$ (*b*-veto category) or
	no requirement ($Z^\prime$ category)
*W*+jets$/t\bar{t}$ fake factor control region	$m_\mathrm{T}(\ell ,E_{\text {T}}^{\text {miss}})>$ 70 (60) $\text {GeV}$ for $\tau _e\tau _{\mathrm {had}}$ ($\tau _\mu \tau _{\mathrm {had}}$),
	different $\tau _{\mathrm {had-vis}}$ identification for anti-$\tau _{\mathrm {had}}$ sub-region
$t\bar{t}$ validation region	$N_{b\text {-tag}}\ge 1$, $m_\mathrm{T}(\ell ,E_{\text {T}}^{\text {miss}}) > 100~\text {GeV}$
Multi-jet fake factor control region	Invert $e,\mu$ isolation requirement,
	different $\tau _{\mathrm {had-vis}}$ identification for anti-$\tau _{\mathrm {had}}$ sub-region
Multi-jet control region for	$m_\mathrm{T}(\ell ,E_{\text {T}}^{\text {miss}})<$ 30 $\text {GeV}$, no $e,\mu$ isolation requirement,
$r_{\text {MJ}}$ estimation	no $\tau _{\mathrm {had-vis}}$ passing loose identification,
	$N_{\text {jet}}\ge 1$ (*b*-veto category), $N_{\text {jet}} \ge 2$ (*b*-tag category)
Control region for correction of electrons	$80< m_{e,\tau } < 110~\text {GeV}$ for 1-track $\tau _{\mathrm {had-vis}}$
misidentified as $\tau _{\mathrm {had-vis}}$	$90< m_{e,\tau } < 100~\text {GeV}$ for 3-track $\tau _{\mathrm {had-vis}}$
$\tau _{\mathrm {had}}\tau _{\mathrm {had}}$ selection	$\Delta \phi ({\tau _{\mathrm {had-vis}}}_{,1}, {\tau _{\mathrm {had-vis}}}_{,2}) > 2.7$,
	$N_{b\text {-tag}}\ge 1$ (*b*-tag category), $N_{b\text {-tag}}= 0$ (*b*-veto category) or
	no requirement ($Z^\prime$ category)
Multi-jet fake factor control region	Pass single-jet trigger, leading $\tau _{\mathrm {had-vis}}$ fails medium identification,
	no tracks, nor charge requirements for leading $\tau _{\mathrm {had-vis}}$,
	$\frac{p_{\text {T}} ^{{\tau _{\mathrm {had-vis}}}_{,2}}}{p_{\text {T}} ^{{\tau _{\mathrm {had-vis}}}_{,1}}} > 0.3$, no $\Delta \phi ({\tau _{\mathrm {had-vis}}}_{,1}, {\tau _{\mathrm {had-vis}}}_{,2})$ requirement
Fake-rate control region	Pass single-muon trigger, isolated muon with $p_{\text {T}} > 55~\text {GeV}$,
	$\tau _{\mathrm {had-vis}}$ with $p_{\text {T}} > 50~\text {GeV}$, $\Delta \phi (\mu ,\tau _{\mathrm {had-vis}})>2.4$,
	$\sum _{L=\mu ,\tau }\cos \Delta \phi (L,E_{\text {T}}^{\text {miss}})<0$ (for *b*-veto category only)
$W\rightarrow \mu \nu$ control region for	Pass single-muon trigger, isolated muon with $p_{\text {T}} > 110~\text {GeV}$,
*W*+jets $m_\mathrm{T}^\mathrm{tot}$ correction	$\tau _{\mathrm {had-vis}}$ with $p_{\text {T}} > 55~\text {GeV}$

### $\tau _{\mathrm {lep}}\tau _{\mathrm {had}}$ background estimate

The main backgrounds in the $\tau _{\mathrm {lep}}\tau _{\mathrm {had}}$ channel arise from $Z \rightarrow \tau \tau$ production, followed by processes with a misidentified $\tau _{\mathrm {had-vis}}$ in the *b*-veto category and $t\bar{t}$ production, with either a true $\tau$ lepton or a jet misidentified as a $\tau _{\mathrm {had-vis}}$, in the *b*-tag category.

Background processes where the $\tau _{\mathrm {had}}$ candidate, or both the lepton and $\tau _{\mathrm {had}}$ candidates, arise from misidentified jets are dominated by *W*+jets ($t\bar{t}$) and multi-jet processes, for the *b*-veto (*b*-tag) category. A data-driven “fake factor” (FF) technique is used to estimate the contribution of these processes to the signal region. The fake factors are derived separately for the *b*-veto and *b*-tag categories using fake factor control regions (see Table 1) dominated by a particular background process (Pr), and are defined as:

3 $$\begin{aligned} \text {FF}(\text {Pr}) = \frac{N(\text {nominal} \tau _{\mathrm {had-vis}}\text {ID}, \text {Pr})}{N(\text {anti-} \tau _{\mathrm {had-vis}}\text {ID}, \text {Pr})}, \end{aligned}$$

where $N(\text {nominal}$ $\tau _{\mathrm {had-vis}}$ $\text {ID}, \text {Pr})$ is the number of $\tau _{\mathrm {had-vis}}$ candidates in data satisfying the “medium” $\tau _{\mathrm {had-vis}}$ identification criterion and $N(\text {anti-}$ $\tau _{\mathrm {had-vis}}$ $\text {ID}, \text {Pr})$ is the number of $\tau _{\mathrm {had-vis}}$ candidates failing this criterion but meeting a loose requirement on the BDT score. The latter requirement defines the “anti-$\tau _{\mathrm {had}}$” sub-region, which selects the same kind of objects mimicking $\tau _{\mathrm {had-vis}}$ candidates as those fulfilling the identification criteria. The true $\tau _{\mathrm {had}}$ contamination in the fake factor control regions is subtracted using simulation. In all the control regions, the fake factors are parameterized as a function of the transverse momentum and number of tracks of the reconstructed $\tau _{\mathrm {had-vis}}$ object.

The fake factor for *W*+jets and $t\bar{t}$ backgrounds, FF(*W*+jets$/t\bar{t})$, is measured in a fake factor control region that is identical to the signal region, except that the $m_\mathrm{T}(\ell ,E_{\text {T}}^{\text {miss}})$ selection criterion is reversed to $m_\mathrm{T}(\ell ,E_{\text {T}}^{\text {miss}})> 60\,(70)~\text {GeV}$ for the $\tau _{\mu }\tau _{\mathrm {had}}$ ($\tau _{e}\tau _{\mathrm {had}}$) channel. The purity of the *W*+jets background in the *b*-veto category of the control region is about $95\%$, while in the *b*-tag category both the *W*+jets and $t\bar{t}$ processes contribute. The fake factor value for the *b*-tag category was found to be compatible with the value corresponding to the *b*-veto category. To improve the statistical precision, the fake factor measured in a control region without requirements on the number of *b*-tags is used for the *b*-tag category. The same fake factor is used in the search for the $Z^\prime$ boson. For multi-jet events (MJ), the fake factor FF$(\text {MJ})$ is measured in a fake factor control region defined by inverting the isolation requirement on the electron or muon. The purity of multi-jet events in this control region exceeds 99 %. The fake factors are derived separately for the *b*-veto and *b*-tag categories by requiring no *b*-tag and at least one *b*-tag, respectively. For the $Z^{\prime }$ analysis, no *b*-tag requirement is considered.

The shapes and normalization of background contributions in the signal region are then estimated by applying these fake factors to events that pass the anti-$\tau _{\mathrm {had}}$ region selection but otherwise satisfy all signal region requirements. In this analysis, the fake factors are combined and weighted by the predicted contribution of each background process to the anti-$\tau _{\mathrm {had}}$ region:

4 $$\begin{aligned} \text {FF}(\text {comb}) = \text {FF}(W+\text {jets}/t\bar{t}) \times r_{W/t\bar{t}} + \text {FF}(\text {MJ}) \times r_{\text {MJ}}, \end{aligned}$$

where $r_{\text {MJ}}$ denotes the fraction of multi-jet events in the anti-$\tau _{\mathrm {had}}$ region and $r_{W/t\bar{t}} = 1 - r_{\text {MJ}}$. This neglects the differences between the fake factors for *W*+jets/$t\bar{t}$ and other processes, such as *Z* production. The parameter $r_{\text {MJ}}$ is estimated, separately for the *b*-veto and *b*-tag categories, in two steps using a data-driven method. First, the rates at which jets are misidentified as electrons or muons are measured from the ratio of leptons passing and failing the lepton isolation requirement in a region enriched in multi-jet events. This multi-jet control region is defined in Table 1. The predicted multi-jet rate is then applied to events in the anti-$\tau _{\mathrm {had}}$ sub-region that also fail the lepton isolation, in order to calculate $r_{\text {MJ}}$ as a function of $\tau _{\mathrm {had-vis}}$ $p_{\text {T}}$ separately for the $\tau _e\tau _{\mathrm {had}}$ and $\tau _\mu \tau _{\mathrm {had}}$ channels. When the fake factor is applied to the anti-$\tau _{\mathrm {had}}$ sub-region events, the contributions from correctly identified $\tau _{\mathrm {had-vis}}$ and from electrons and muons misidentified as $\tau _{\mathrm {had-vis}}$ candidates are subtracted using the default MC simulation described in Sect. 2.

Background processes where the electron or the muon is identified as a $\tau _{\mathrm {had-vis}}$ object are modelled using simulation. The main source of such backgrounds is $Z (\rightarrow ee)$+jets events in the $\tau _{e}\tau _{\mathrm {had}}$ channel, which are reduced using the $m_{\text {vis}}(e,\tau _{\mathrm {had}})$ mass-window veto described in Sect. 4.1. To account for mismodelling of electrons misidentified as $\tau _{\mathrm {had-vis}}$ objects in $Z\rightarrow ee$+jets events, the simulation is corrected as a function of the lepton $\eta$ using data control regions defined by reversing the mass-window criterion, as listed in Table 1. The correction amounts to 15 % for three-track $\tau _{\mathrm {had-vis}}$, while for the one-track $\tau _{\mathrm {had-vis}}$ objects the correction varies from 20 % in the barrel region to up to 200 % in the end-cap region.

**Fig. 3:**
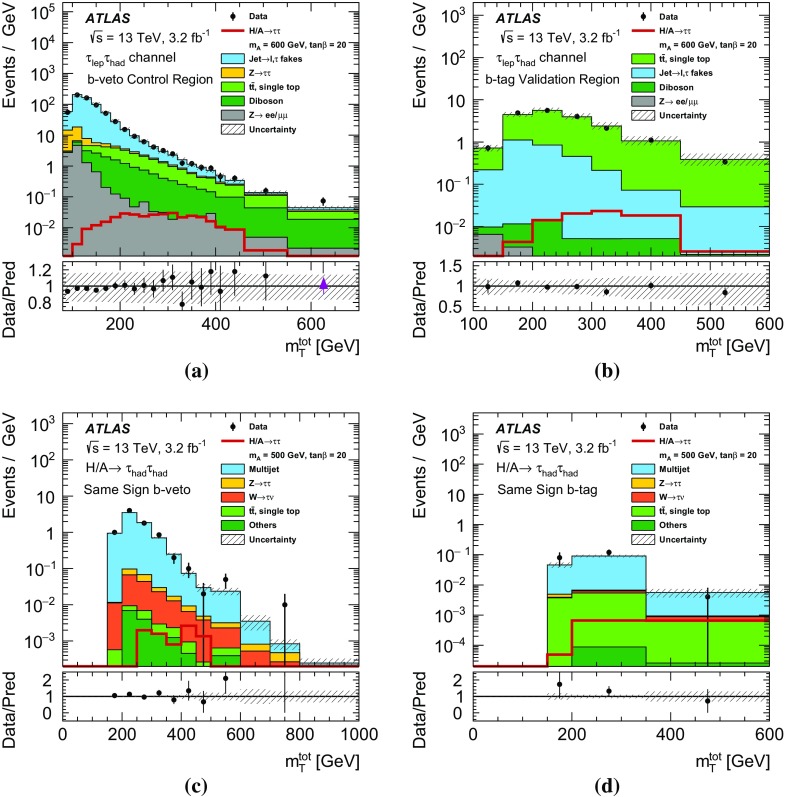
The distributions of $m_\mathrm{T}^{\text {tot}}$ in **a** the $\tau _{\mathrm {lep}}\tau _{\mathrm {had}}$ channel *W*+jets control region, **b** the $t\bar{t}$ validation region of the $\tau _{\mathrm {lep}}\tau _{\mathrm {had}}$ channel, **c** the $\tau _{\mathrm {had}}\tau _{\mathrm {had}}$ channel *b*-veto same-sign validation region and **d** the $\tau _{\mathrm {had}}\tau _{\mathrm {had}}$ channel *b*-tag same-sign validation region. The various control and validation regions are defined in Table 1. The data are compared to the background prediction and a hypothetical MSSM $H/A\rightarrow \tau \tau$ signal ($m_A = 500~\text {GeV}$ and $\tan \beta = 20$). The Monte Carlo statistics of the signal is limited in the background-dominated regions. The label “Others” in **c** and **d** refers to contributions due to diboson, $Z(\rightarrow \ell \ell )$+jets and $W(\rightarrow \ell \nu )$+jets production. The background uncertainty includes statistical and systematic uncertainties. The bins have a varying size and overflows are included in the last bin of the distributions

The $m_\mathrm{T}^{\text {tot}}$ distributions in the $\tau _{\mathrm {lep}}\tau _{\mathrm {had}}$ channel are shown in Fig. 3a, b for the *W*+jets control region and the $t\bar{t}$ validation region, respectively. The latter is identical to the *b*-tag category definition, except for the $m_\mathrm{T}(\ell ,E_{\text {T}}^{\text {miss}})$ requirement, which is reversed to $m_\mathrm{T}(\ell ,E_{\text {T}}^{\text {miss}}) > 100~\text {GeV}$.

### $\tau _{\mathrm {had}}\tau _{\mathrm {had}}$ background estimate

The dominant background process for the $\tau _{\mathrm {had}}\tau _{\mathrm {had}}$ channel is multi-jet production, the cross section of which is several orders of magnitude higher than that of the signal processes. Despite the large suppression of this background thanks to the event selection, a sizeable contribution of events with two jets misidentified as $\tau _{\mathrm {had-vis}}$ candidates remains. A fake factor technique is used to normalize and model this background. Fake factors parameterized as a function of $p_{\text {T}} (\tau _{\mathrm {had-vis}})$ and the number of tracks are derived from a control region enriched with multi-jet events, described in Table 1. The factors are derived independently for the *b*-tag and *b*-veto categories in the search for *H* / *A* bosons, and inclusively in the search for $Z^\prime$ bosons. They are then applied to data events where the leading $\tau _{\mathrm {had-vis}}$ has passed the $\tau _{\mathrm {had-vis}}$ identification requirement in the signal region, while the sub-leading $\tau _{\mathrm {had-vis}}$ candidate has passed only a loose requirement on the BDT score. The contributions from non-multi-jet production processes are subtracted using simulation.

For $t\bar{t}$ and *W*+jets events, along with other simulated background processes, the probability of a jet being misidentified as a $\tau _{\mathrm {had-vis}}$ is modelled with a “fake-rate” technique. The rates of jets being misidentified as a $\tau _{\mathrm {had-vis}}$ are measured from data as a function of the transverse momentum and number of tracks of the reconstructed $\tau _{\mathrm {had-vis}}$. The fake-rate control regions are described in Table 1 and are enriched in $W(\rightarrow \mu \nu )$+jets for the *b*-veto category and $t\bar{t}$ events for the *b*-tag category,

The fake rate is then applied to the simulated events as a weight for each of the reconstructed $\tau _{\mathrm {had-vis}}$ that does not match geometrically a true $\tau$ lepton. Fake rates derived in the fake-rate control region of the *b*-tag category are used for simulated $t\bar{t}$ and single top quark events, while fake rates obtained in the *b*-veto control region are applied to the remaining processes. An additional weight is applied to $W\rightarrow \tau \nu$+jets events as a function of $m_\mathrm{T}^\mathrm{tot}$, to improve the modelling of the kinematics of the *W*+jets simulated events. The reweighting function is derived by fitting the ratio of the data to the simulation for the $W(\rightarrow \mu \nu )\mathrm{+jets}$ process in an additional $W\rightarrow \mu \nu$ control region, defined in analogy with the inclusive signal selection and described in Table 1.

A same-sign validation region, enriched with events where at least one jet is misidentified as a $\tau _{\mathrm {had-vis}}$, is obtained by inverting the opposite-sign requirement of the two $\tau _{\mathrm {had-vis}}$ candidates. Distributions of $m_\mathrm{T}^{\text {tot}}$ in the $\tau _{\mathrm {had}}\tau _{\mathrm {had}}$ channel same-sign validation region are shown in Fig. 3c, d. The good performance multi-jet background estimation method is demonstrated by the agreement of the data with the background prediction.

## Systematic uncertainties

The signal efficiency and the background estimates are affected by uncertainties associated with the detector simulation, the signal modelling and the data-driven background determination.

The integrated luminosity measurement has an uncertainty of 5 % and is used for all MC samples. Uncertainties related to the detector are included for all signal and backgrounds that are estimated using simulated samples. These uncertainties are also taken into account for simulated samples that are used in the derivation of data-driven background estimates. All instrumental systematic uncertainties arising from the reconstruction, identification and energy scale of $\tau _{\mathrm {had-vis}}$ candidates, electrons, muons, (*b*-)jets and the soft term of the $E_{\text {T}}^{\text {miss}}$ measurement are considered. The effect of the energy scale uncertainties on the objects is propagated to the $E_{\text {T}}^{\text {miss}}$ determination. The electron, muon, jet and $E_{\text {T}}^{\text {miss}}$ systematic uncertainties described above are found to have a very small effect.

Systematic uncertainties resulting from the data-driven background estimates are derived as follows. In the $\tau _{\mathrm {lep}}\tau _{\mathrm {had}}$ channel, the combined fake-factor method includes uncertainties in the *W*+jets$/t\bar{t}$ fake factors, the multi-jet fake factors, and the $r_{\text {MJ}}$ estimation. For the *W*+jets fake factors the main uncertainties arise from the dependence on $\Delta \phi (\tau _{\mathrm {had-vis}},E_{\text {T}}^{\text {miss}})$, from the difference between the relative contributions of quark- and gluon-initiated jets faking $\tau _{\mathrm {had-vis}}$ in the control region and the signal region, and from the contamination by backgrounds other than *W*+jets in the control region, which are estimated using simulation. The uncertainty is parameterized as a function of the anti-$\tau _{\mathrm {had-vis}}$ $p_{\text {T}}$ and amounts approximately to 17 % for jets misidentified as one-track $\tau$ candidates and varies between 16 and 34 % for jets misidentified as three-track $\tau$ candidates. Uncertainties related to non-*W*+jets events were studied and have no significant impact on the fake-factor determination. For the multi-jet fake factors and $r_{\text {MJ}}$, the uncertainty is dominated by the number of data events in the control region and the subtraction of the remaining non-multi-jet backgrounds using simulation. Typical values of the total uncertainties for $r_{\text {MJ}}$ are between 7 and 20 % and for the multi-jet fake factors between 10 and 20 %, depending on the channel and the $\tau _{\mathrm {had-vis}}$ candidate $p_{\text {T}}$. In addition, the effect on the background estimate due to the anti-$\tau _{\mathrm {had}}$ region definition is examined. The loose $\tau _{\mathrm {had-vis}}$ identification requirement used in the definition of this region is varied to estimate the corresponding uncertainty, which is 5 and 1 % in the $\tau _e\tau _{\mathrm {had}}$ and the $\tau _\mu \tau _{\mathrm {had}}$ channel, respectively.

In the $\tau _{\mathrm {had}}\tau _{\mathrm {had}}$ channel, the uncertainty in the fake-factor measurement used for the multi-jet background estimation is obtained as the sum in quadrature of the statistical uncertainty of the measurement and the difference between the fake factors determined from same-sign and from opposite-sign events. The fake rates for jets misidentified as $\tau _{\mathrm {had-vis}}$ are determined from data. The main systematic uncertainty arises from the statistical uncertainty of the fake-rate measurement and it ranges from 7 to 30 % as a function of the $\tau _{\mathrm {had-vis}}$ $p_{\text {T}}$. In the $\tau _{\mathrm {had}}\tau _{\mathrm {had}}$ channel, the uncertainty in the parameters of the function used to reweight the $W\rightarrow \tau \nu$+jets background is propagated to the $m_\mathrm{T}^\mathrm{tot}$ distribution, where its effect ranges from 5 to 20 %.

Theoretical cross-section uncertainties are considered for all backgrounds estimated using simulation. For *Z*+jets and diboson production, uncertainties of $5\%$ and $6\%$ are used, respectively, combining PDF$+\alpha _{\text {S}}$ and scale variation uncertainties in quadrature. For $t\bar{t}$ [102] and single top-quark [123, 124] production, a $6\%$ uncertainty is assigned based on scale, PDF and top-quark mass uncertainties. Additional uncertainties related to initial- and final-state radiation modelling, tune and (for $t\bar{t}$ only) the choice of the hdamp parameter value in POWHEG-BOX v2, which controls the amount of radiation produced by the parton shower, were also taken into account [125]. The uncertainty in the fragmentation model is evaluated by comparing $t\bar{t}$ events generated with POWHEG-BOX v2 interfaced to either HERWIG++ [126] or PYTHIA6. The POWHEG+HERWIG++ and aMC@NLO+HERWIG++ generators are used to estimate the uncertainty in generating the hard scatter. The variation of the *b*-tag category acceptance for these uncertainties is from $-10$ to $+30\,\%$ ($-33$ to $+38\,\%$) in the $\tau _{\mathrm {lep}}\tau _{\mathrm {had}}$ ($\tau _{\mathrm {had}}\tau _{\mathrm {had}}$) channel.

Uncertainties related to signal modelling are discussed in the following. Uncertainties due to the factorization and renormalization scale choices are estimated from the effect on the signal acceptance of doubling or halving these factors either coherently or oppositely. Uncertainties due to the initial- and final-state radiation, as well as multiple parton interaction for the signal are also taken into account. These uncertainties are estimated from the PYTHIA8 A14 tune [61] for the *b*-associated production and the AZNLO PYTHIA8 tune [60] for the gluon–gluon fusion production. The envelope of the variations resulting from the use of the alternative PDFs in the PDF4LHC15$\_$nlo$\_$100 [127] set is used in order to estimate the PDF uncertainty for gluon–gluon fusion production. For the *b*-associated production uncertainty, a comparison among NNPDF30_nlo_as_0118_nf_4 [127], CT14nlo_NF4 [58], MSTW2008nlo68cl_nf4 [128] and CT10_nlo_nf4 [57] PDF sets is employed. Since no statistically significant effect on the shape of the reconstructed mass distribution is observed, each contribution is taken solely as a normalization uncertainty. The total uncertainty ranges between 15 and 25 %.

## Results

The parameter of interest is the signal strength, $\mu$. It is defined as the ratio of the observed to the predicted value of the cross section times branching fraction, where the prediction is evaluated for a particular MSSM or $Z^\prime$ assumption. Hence, the value $\mu =0$ corresponds to the absence of a signal, whereas the value $\mu =1$ indicates the presence of a signal as predicted by the theoretical model under study. To estimate $\mu$, a likelihood function constructed as the product of Poisson probability terms is used. Signal and background predictions depend on systematic uncertainties, which are parameterized as nuisance parameters and are constrained using Gaussian probability distributions. For the MSSM Higgs boson search a binned likelihood function is constructed in bins of the $m_\mathrm{T}^\mathrm{tot}$ distributions, chosen to ensure sufficient background statistics in each bin. The search for a $Z^\prime$ boson is a counting experiment, summing the number of events above a certain $m_\mathrm{T}^{\text {tot}}$ threshold. The threshold is chosen for each $Z^\prime$ mass hypothesis to maximize the expected significance and ranges from 400 $\text {GeV}$ at low $Z^\prime$ mass to 750 $\text {GeV}$ at high $Z^\prime$ mass. The asymptotic approximation is used with the test statistic $\tilde{q}_\mu$ [129] to test the compatibility of the data with the assumed signal.

**Fig. 4:**
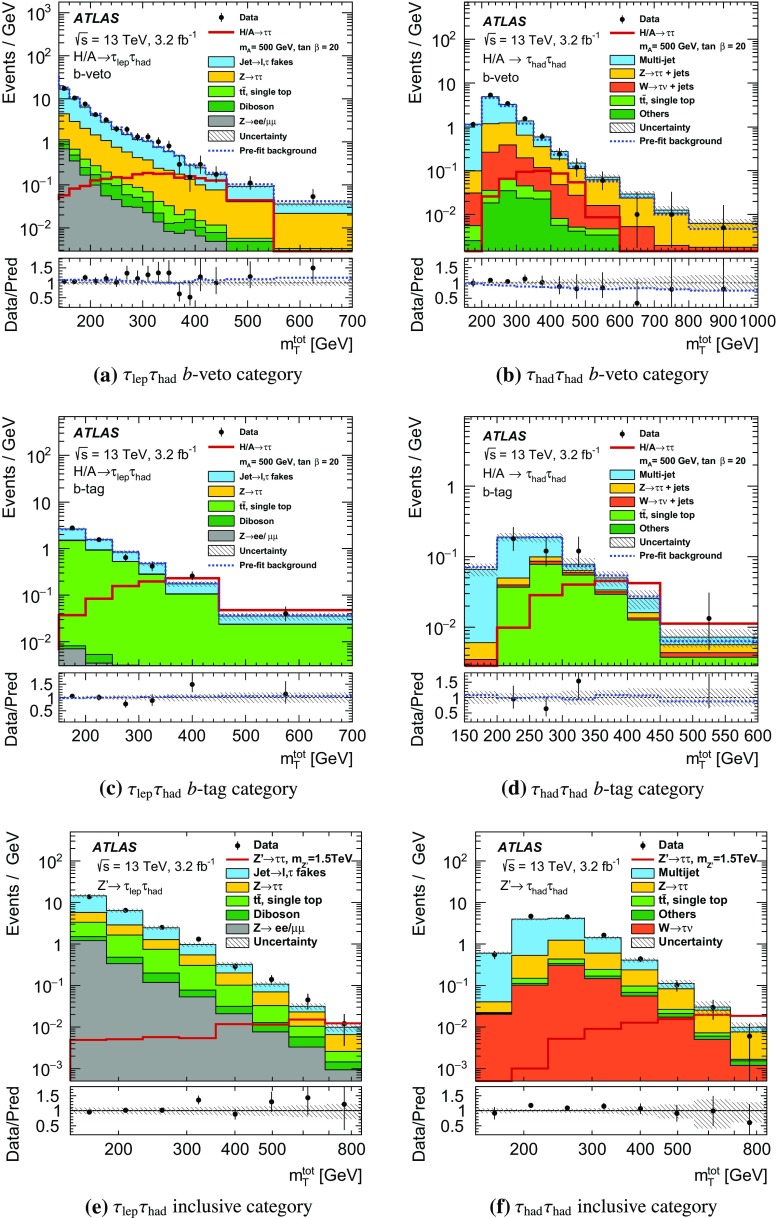
The distribution of $m_\mathrm{T}^{\text {tot}}$ for the *b*-veto category of the **a** $\tau _{\mathrm {lep}}\tau _{\mathrm {had}}$ and **b** $\tau _{\mathrm {had}}\tau _{\mathrm {had}}$ channels, the *b*-tag category of the **c** $\tau _{\mathrm {lep}}\tau _{\mathrm {had}}$ and **d** $\tau _{\mathrm {had}}\tau _{\mathrm {had}}$ channels, and the inclusive category of the **e** $\tau _{\mathrm {lep}}\tau _{\mathrm {had}}$ and **f** $\tau _{\mathrm {had}}\tau _{\mathrm {had}}$ channels. The label “Others” in **b**, **d** and **f** refers to contributions due to diboson, $Z(\rightarrow \ell \ell )$+jets and $W(\rightarrow \ell \nu )$+jets production. For the *b*-veto and *b*-tag categories, the binning displayed is that entering into the statistical fit discussed in Sect. 7, while the predictions and uncertainties for the background and signal processes are obtained from the fit under the hypothesis of no signal. The inclusive category distributions are shown before any statistical fit. Overflows are included in the last bin of the distributions

The number of observed $\tau _{\mathrm {lep}}\tau _{\mathrm {had}}$ and $\tau _{\mathrm {had}}\tau _{\mathrm {had}}$ data events, along with the predicted event yields from background and signal processes, in the signal regions are shown in Table 2. The observed event yields are compatible with the expected event yield from SM processes, within uncertainties. The $m_\mathrm{T}^{\text {tot}}$ mass distributions are shown in Fig. 4. The results from the $\tau _{\mathrm {lep}}\tau _{\mathrm {had}}$ and $\tau _{\mathrm {had}}\tau _{\mathrm {had}}$ channels are combined to improve the sensitivity to *H* / *A* and $Z^\prime$ boson production.

**Table 2:** Observed number of events and background predictions in the *b*-tag and *b*-veto categories for the $\tau _{e}\tau _{\mathrm {had}}$, $\tau _{\mu }\tau _{\mathrm {had}}$ and $\tau _{\mathrm {had}}\tau _{\mathrm {had}}$ channels. The background predictions and uncertainties are obtained from the statistical procedure discussed in Sect. 7. In the $\tau _{\mathrm {lep}}\tau _{\mathrm {had}}$ channel, the processes other than “Jet $\rightarrow \ell , \tau _{\mathrm {had-vis}}$ fakes” require a true hadronically decaying $\tau$ lepton or an electron or muon misidentified as a $\tau _{\mathrm {had-vis}}$. The expected signal yields for the $m_{h}^{\text {mod}+}$ scenario are shown for comparison

	*b*-tag category	*b*-veto category
$\tau _{e}\tau _{\mathrm {had}}$ channel		
$Z\rightarrow \tau \tau$+jets	42 ± 7	4500 ± 250
Jet $\rightarrow \ell , \tau _{\mathrm {had-vis}}$ fakes	128 ± 18	5400 ± 350
$Z\rightarrow \ell \ell$+jets	3.6 ± 1.5	590 ± 120
$t\bar{t}$ and single top quark	115 ± 16	35 ± 5
Diboson	0.33 ± 0.07	44 ± 4
Total prediction	289 ± 24	10600 ± 360
Data	275	10619
*ggH* $m_{A}=500 \text {GeV}, \tan \beta =20$	0.020 ± 0.010	1.2 ± 0.2
*bbH* $m_{A}=500 \text {GeV}, \tan \beta =20$	6.4 ± 1.7	7.4 ± 1.9
$\tau _{\mu }\tau _{\mathrm {had}}$ channel		
$Z\rightarrow \tau \tau$+jets	42 ± 6	5500 ± 300
Jet $\rightarrow \ell , \tau _{\mathrm {had-vis}}$ fakes	109 ± 14	2760 ± 170
$Z\rightarrow \ell \ell$+jets	5.2 ± 0.6	830 ± 50
$t\bar{t}$ and single top quark	136 ± 15	40 ± 5
Diboson	0.34 ± 0.07	55 ± 5
Total prediction	293 ± 19	9200 ± 300
Data	312	9163
*ggH* $m_{A}=500 \text {GeV}, \tan \beta =20$	0.016 ± 0.005	1.1 ± 0.2
*bbH* $m_{A}=500 \text {GeV}, \tan \beta =20$	3.3 ± 1.3	6.4 ± 1.7
$\tau _{\mathrm {had}}\tau _{\mathrm {had}}$ channel		
$Z\rightarrow \tau \tau$+jets	1.9 ± 0.3	146 ± 20
Multi-jet	17 ± 3	396 ± 16
$W\rightarrow \tau \nu$+ jets	1.1 ± 0.2	45 ± 7
$t\bar{t}$ and single top quark	11 ± 3	4.5 ± 0.9
Others	0.13 ± 0.03	6.3 ± 0.8
Total prediction	31 ± 4	598 ± 21
Data	23	628
*ggH* $m_{A}=500 \text {GeV}, \tan \beta =20$	0.034 ± 0.014	2.2 ± 0.7
*bbH* $m_{A}=500 \text {GeV}, \tan \beta =20$	8 ± 3	15 ± 5

The fractional contributions of the most important sources of systematic uncertainty to the total uncertainty in the signal cross-section measurement are shown for two signal assumptions: Table 3(top) represents an MSSM Higgs boson hypothesis ($m_A=500$ $\text {GeV}$, $\tan \beta =20$) and Table 3(bottom) corresponds to an SSM $Z^\prime$ boson hypothesis ($m_{Z^\prime }=1.75$  ${\mathrm {TeV}}$). As shown in this table, the sensitivity of the search is limited by statistical uncertainties.

**Table 3:** Fractional impact of the most important sources of systematic uncertainty on the total uncertainty of the signal strength, for (top) the MSSM signal hypothesis of $m_A=500$ $\text {GeV}$, $\tan \beta =20$, (bottom) the $Z^\prime _\mathrm {SSM}$ signal hypothesis of $m_{Z^\prime }=1.75$  ${\mathrm {TeV}}$. For each source of uncertainty, $F_{\pm } = \pm \frac{\sigma ^{2}_{\text {source}}}{\sigma ^{2}_{\text {total}}}$ is defined as the positive (negative) fractional contribution to the signal strength uncertainty

Source of uncertainty	$F_{-}$ (%)	$F_{+}$ (%)
MSSM analysis		
$t\bar{t}$ and single-top-quark backgrounds normalization	$-13\phantom {.0}$	$+11\phantom {.0}$
$\tau _{\mathrm {had-vis}}$ energy scale	$-\phantom {0}3\phantom {.0}$	$+\phantom {0}8\phantom {.0}$
$\tau _{\mathrm {had}}$ trigger	$-\phantom {0}0.5$	$+10\phantom {.0}$
Signal acceptance	$-\phantom {0}6\phantom {.0}$	$+\phantom {0}1.9$
Jet-to-$\tau _{\mathrm {had-vis}}$ fake rate ($\tau _{\mathrm {lep}}\tau _{\mathrm {had}}$)	$-\phantom {0}1.5$	$+\phantom {0}2.4$
Multi-jet background ($\tau _{\mathrm {had}}\tau _{\mathrm {had}}$)	$-\phantom {0}0.4$	$+\phantom {0}0.3$
$t\bar{t}$ background modelling	$-\phantom {0}0.1$	$+\phantom {0}1.0$
Jet-to-$\tau _{\mathrm {had-vis}}$ fake rate ($\tau _{\mathrm {had}}\tau _{\mathrm {had}}$)	$-\phantom {0}0.2$	$+\phantom {0}0.2$
Jet-to-vertex association	$-\phantom {0}0.1$	$+\phantom {0}0.1$
Statistics (data and simulation)	$-75\phantom {.0}$	$+65\phantom {.0}$
$Z^\prime _\mathrm {SSM}$ analysis		
$\tau _{\mathrm {had-vis}}$ energy scale	$-13\phantom {.000}$	$+\phantom {0}5\phantom {.00}$
Pile-up	$-\phantom {0}0.015$	$+\phantom {0}1.3\phantom {0}$
$Z+$jets backgrounds cross section and acceptance	$-\phantom {0}0.3\phantom {00}$	$+\phantom {0}0.4\phantom {0}$
Signal PDF	$-\phantom {0}0.3\phantom {00}$	$+\phantom {0}0.5\phantom {0}$
Jet-to-$\tau _{\mathrm {had-vis}}$ fake rate ($\tau _{\mathrm {had}}\tau _{\mathrm {had}}$)	$-\phantom {0}0.3\phantom {00}$	$+\phantom {0}0.3\phantom {0}$
$\tau _{\mathrm {had-vis}}$ identification	$-\phantom {0}0.19\phantom {0}$	$+\phantom {0}0.18$
Luminosity	$-\phantom {0}0.16\phantom {0}$	$+\phantom {0}0.15$
$W\rightarrow \tau \nu$+jets background reweighting ($\tau _{\mathrm {had}}\tau _{\mathrm {had}}$)	$-\phantom {0}0.17\phantom {0}$	$+\phantom {0}0.10$
Statistics (data and simulation)	$-85\phantom {.000}$	$+92\phantom {.00}$

**Fig. 5:**
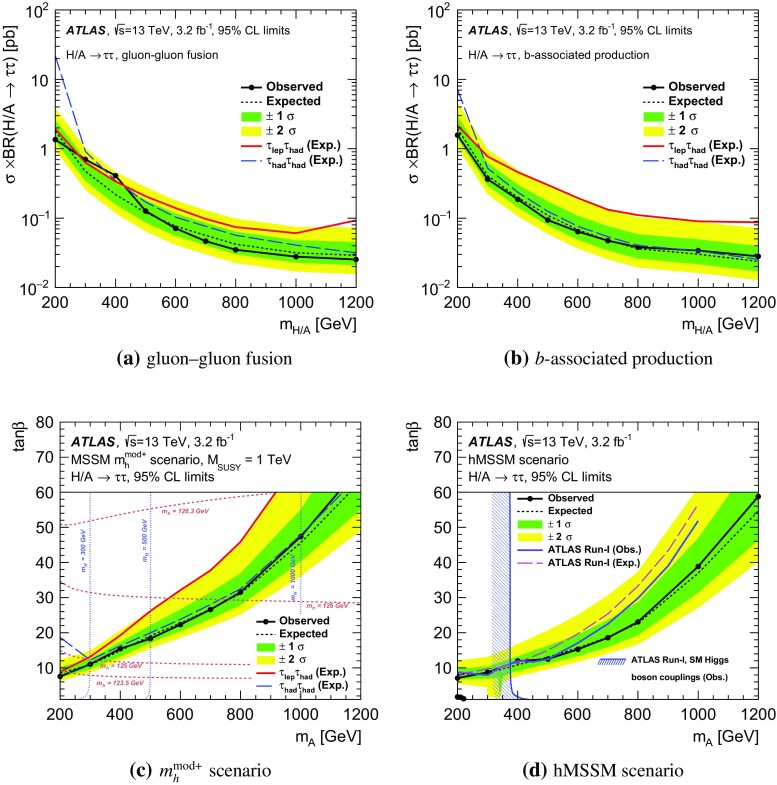
The observed and expected 95 % CL upper limits on the production cross section times branching fraction of a scalar particle are shown for the combination of the $\tau _{\mathrm {lep}}\tau _{\mathrm {had}}$ and the $\tau _{\mathrm {had}}\tau _{\mathrm {had}}$ channels. The production mechanism of $H/A\rightarrow \tau \tau$ is assumed to be **a** gluon–gluon fusion or **b** *b*-associated production. For comparison, the expected limits for the individual channels, $\tau _{\mathrm {lep}}\tau _{\mathrm {had}}$ and $\tau _{\mathrm {had}}\tau _{\mathrm {had}}$, are shown as well. The observed and expected 95 % CL limits on $\tan \beta$ as a function of $m_A$ are shown in **c** for the MSSM $m_h^{\text {mod+}}$ scenario and **d** for the hMSSM scenario. For comparison, the expected limits from the individual channels, $\tau _{\mathrm {lep}}\tau _{\mathrm {had}}$ and $\tau _{\mathrm {had}}\tau _{\mathrm {had}}$, are given in **c**, while the observed and expected limits from the ATLAS Run-1 analysis in Ref. [28] are shown in d. Dashed lines of constant $m_h$ and $m_H$ are shown in red and blue, respectively

The data are found to be in good agreement with the predicted background yields and hence the results are given as exclusion limits. These are set using the modified frequentist method known as CL$_{s}$ [130]. Observed and expected 95 % confidence level (CL) upper limits on the cross section times branching fraction for the production of a single scalar boson *H* / *A* decaying to $\tau \tau$, as a function of the mass of the boson $m_{H/A}$, are shown in Fig. 5a, b. The limits are calculated for both the gluon–gluon fusion and *b*-associated production modes, using a combination of the $\tau _{\mathrm {lep}}\tau _{\mathrm {had}}$ and $\tau _{\mathrm {had}}\tau _{\mathrm {had}}$ channels and assuming the natural width of the boson to be negligible compared to the experimental resolution (as expected over the probed MSSM parameter space). The lowest excluded cross section times branching fraction values range from $\sigma \times BR = 1.4~\mathrm {pb}$ at $m_{H/A} = 200~\text {GeV}$ to $\sigma \times BR = 0.025~\mathrm {pb}$ at $m_{H/A} = 1.2~~{\mathrm {TeV}}$ for a scalar boson produced via gluon–gluon fusion. Similarly, for the *b*-associated production mechanism the lowest excluded values range is from $\sigma \times BR = 1.6~\mathrm {pb}$ at $m_{H/A} = 200\,\text {GeV}$ to $\sigma \times BR = 0.028~\mathrm {pb}$ at $m_{H/A} = 1.2\,~{\mathrm {TeV}}$.

The observed and expected 95 % CL limits on $\tan \beta$ as a function of $m_A$, for the combination of $\tau _{\mathrm {lep}}\tau _{\mathrm {had}}$ and $\tau _{\mathrm {had}}\tau _{\mathrm {had}}$ channels in the MSSM $m_h^{\text {mod+}}$ and hMSSM scenarios are shown in Fig. 5c, d. The expected limit in the $m_h^{\text {mod+}}$ scenario is compared to the expected limits from the individual $\tau _{\mathrm {lep}}\tau _{\mathrm {had}}$ and $\tau _{\mathrm {had}}\tau _{\mathrm {had}}$ channels. For the $m_h^{\text {mod+}}$ figure, lines of constant $m_h$ and $m_H$ are shown. For the hMSSM scenario, the exclusion arising from the SM Higgs boson coupling measurements of Ref. [131] is also shown, in addition to the ATLAS Run-1 $H/A\rightarrow \tau \tau$ search result of Ref. [28]. The $\tan \beta$ constraints in the hMSSM scenario are stronger than those in the $m_h^{\text {mod+}}$ scenario. This is due to the presence of low-mass neutralinos in the $m_h^{\text {mod+}}$ scenario that reduce the $H/A\rightarrow \tau \tau$ branching fraction and which are absent in the hMSSM scenario. In the hMSSM scenario, the most stringent constraints on $\tan \beta$ for the combined search exclude $\tan \beta > 7.1$ for $m_A = 200~\text {GeV}$ and $\tan \beta > 39$ for $m_A = 1~~{\mathrm {TeV}}$ at the 95 % CL. In the MSSM $m_h^{\text {mod+}}$ scenario, the 95 % CL upper limits exclude $\tan \beta > 7.6$ for $m_A = 200~\text {GeV}$ and $\tan \beta > 47$ for $m_A = 1~~{\mathrm {TeV}}$. The feature of the expected limits in the hMSSM scenario exclusion plot at around $m_A = 350$ $\text {GeV}$ is due to the behaviour of the branching ratio $A\rightarrow \tau \tau$ close to the $A\rightarrow t\bar{t}$ kinematic threshold. Some sensitivity of the search is also expected around $\tan \beta \sim 1$, $m_A\sim 200$ $\text {GeV}$ due to the increase of the gluon–gluon fusion cross section induced by the increased coupling to the top quark.

**Fig. 6:**
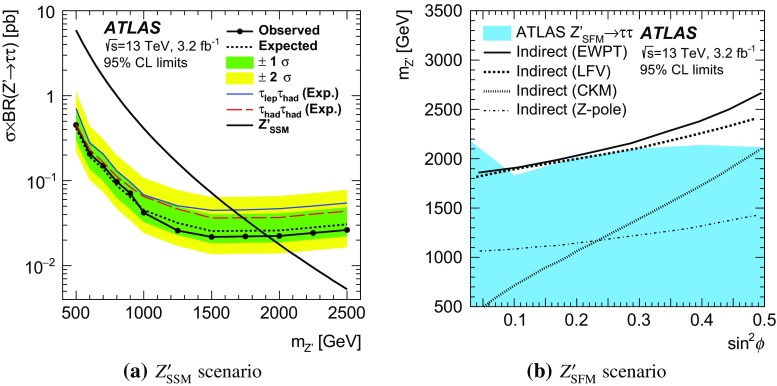
The 95 % CL upper limit on the cross section times branching fraction for a $Z'\rightarrow \tau \tau$ in **a** the Sequential Standard Model and 95% CL exclusion on **b** the SFM parameter space, overlaid with indirect limits at 95 % CL from fits to electroweak precision measurements [132], lepton flavour violation [133], CKM unitarity [134] and *Z*-pole measurements [41]

In the search for the $Z^\prime$ boson, the observed number of events in the signal regions of the $\tau _{\mathrm {lep}}\tau _{\mathrm {had}}$ and $\tau _{\mathrm {had}}\tau _{\mathrm {had}}$ channels are consistent with the SM predictions. The resulting 95% CL upper limits are set on the cross section times branching fraction as a function of the mass and shown in Fig. 6a. These results are interpreted in the context of the SSM and SFM in Fig. 6a, b, respectively. The resulting observed (expected) lower limit on the mass of the $Z'_\mathrm{SSM}$ boson is $1.90{}$ ($1.84$)  ${\mathrm {TeV}}$. In the search for the $Z^\prime _\mathrm {SFM}$ boson, results are presented as a function of $\sin ^{2}\phi {}$, where $\phi$ is the mixing angle between the two SU(2) gauge eigenstates of the model. Masses below 1.82–2.17  ${\mathrm {TeV}}$ are excluded in the range $0.1< \sin ^{2}\phi {} < 0.5$, assuming no $\mu -\tau$ mixing. For the value of $\sin ^{2}\phi {}=0.03$, the lower limit on the mass of a $Z^\prime _\mathrm {SFM}$ boson is 2.12  ${\mathrm {TeV}}$, extending the limits from previous direct and indirect searches by more than 200 $\text {GeV}$.

## Conclusions

A search for neutral Higgs bosons of the minimal supersymmetric standard model (MSSM) and for a $Z^\prime$ gauge boson decaying to a pair of $\tau$ leptons is performed using a data sample corresponding to an integrated luminosity of 3.2 fb$^{-1}$ from proton–proton collisions at $\sqrt{s} = 13$  ${\mathrm {TeV}}$ recorded by the ATLAS detector at the LHC. The search finds no indication of an excess over the expected background in the channels considered. Limits are set at the 95 % CL, which provide constraints in the MSSM parameter space. Model-independent upper limits on the production cross section times the $\tau \tau$ branching fraction of a scalar boson versus its mass, in both the gluon–gluon fusion and *b*-associated production modes, are presented. The upper limits on the cross section times branching fraction range from $1.4~(1.6)~\mathrm {pb}$ at $m_{H/A} = 200\,\text {GeV}$ to $0.025~(0.028)~\mathrm {pb}$ at $m_{H/A} = 1.2\,~{\mathrm {TeV}}$ for a scalar boson produced via gluon–gluon fusion (*b*-associated production). In the context of the MSSM $m_{h}^{\text {mod+}}$ scenario, the most stringent 95 % CL upper limit on $\tan \beta$ for the combined search is $\tan \beta < 7.6$ for $m_A = 200~\text {GeV}$. This analysis extends the limits of the previous searches for the mass range $m_A > 500~\text {GeV}$. The search for a $Z^\prime$ boson is interpreted in the context of the sequential standard model (SSM) and the strong flavour model (SFM). Upper limits at the 95 % CL are set on the cross section times branching fraction as a function of the $Z^\prime$ mass. The observed lower limit on the $Z^\prime$ mass is $1.90{}$  ${\mathrm {TeV}}$ for a $Z'_\mathrm{SSM}$ and ranges from 1.82 to 2.17  ${\mathrm {TeV}}$ as a function of the $\sin ^{2}\phi {}$ parameter for a $Z^\prime _\mathrm {SFM}$.

## Footnotes

1 Throughout this paper the inclusion of charge-conjugate decay modes is implied.

2 ATLAS uses a right-handed coordinate system with its origin at the nominal interaction point (IP) in the centre of the detector and the *z*-axis along the beam pipe. The *x*-axis points from the IP to the centre of the LHC ring, and the *y*-axis points upwards. Cylindrical coordinates $(r,\phi )$ are used in the transverse plane, $\phi$ being the azimuthal angle around the beam pipe. The pseudorapidity is defined in terms of the polar angle $\theta$ as $\eta =-\ln \tan (\theta /2)$. Angular distance is measured in units of $\Delta R \equiv \sqrt{(\Delta \eta )^2 + (\Delta \phi )^2}$.

3 The term “signal selection efficiency” refers to the fraction of signal events decaying to $\tau _{\mathrm {lep}}\tau _{\mathrm {had}}$ or $\tau _{\mathrm {had}}\tau _{\mathrm {had}}$ that are subsequently reconstructed within the detector acceptance and pass the selection requirements.
